# First-Line Systemic Treatment Strategies for Unresectable Hepatocellular Carcinoma: A Systematic Review and Network Meta-Analysis of Randomized Clinical Trials

**DOI:** 10.3389/fonc.2021.771045

**Published:** 2021-12-24

**Authors:** Wenfeng Liu, Bing Quan, Shenxin Lu, Bei Tang, Miao Li, Rongxin Chen, Zhenggang Ren, Xin Yin

**Affiliations:** ^1^ Liver Cancer Institute, Zhongshan Hospital, Fudan University, Shanghai, China; ^2^ National Clinical Research Center for Interventional Medicine, Zhongshan Hospital, Fudan University, Shanghai, China

**Keywords:** unresectable hepatocellular carcinoma, network meta-analysis, first line systemic therapy, randomized clinical trials, systematic review

## Abstract

**Objective:**

Several new first-line treatments were recently approved for unresectable hepatocellular carcinoma (HCC). In this meta-analysis, we compare the efficacy and safety of first-line systemic treatments to provide information for clinical decision making in unresectable HCC.

**Methods:**

Pubmed, Science Direct, Web of Science, Scopus, Ovid MEDLINE, Embase, Google Scholar, the Cochrane Library, EMbase, CNKI, CBM, VIP, and the Wanfang databases, as well as the Cochrane Central Register of Controlled Trails were searched for randomized clinical trials evaluating the efficacy of first-line chemotherapy, molecular targeted therapy, or immunotherapy for unresectable HCC. Hazard ratios with 95% confidence intervals (CIs) were calculated to explore the effects of various treatment options on overall survival (OS) and progression-free survival (PFS), whereas odd ratios with 95% CIs were used for adverse events (AEs) and serious adverse events (SAEs). A network meta-analysis was performed to synthesize data and for direct and indirect comparisons between treatments. The cumulative ranking curve (SUCRA) and P score were used to rank treatments. The risk of bias across studies was assessed graphically and numerically using the funnel plot and Egger’s regression test.

**Results:**

Fifteen studies including 9005 patients were analyzed. Sintilimab plus bevacizumab, atezolizumab plus bevacizumab, and donafenib had better OS outcomes than sorafenib. Sintilimab plus bevacizumab, atezolizumab plus bevacizumab, lenvatinib, and linifanib had better PFS outcomes than sorafenib. The results of network meta-analysis showed that sintilimab plus bevacizumab was associated with the best OS and PFS. Egger’s tests indicated that none of the included studies had obvious publication deviation.

**Conclusion:**

Sintilimab plus bevacizumab showed the best OS and PFS outcomes with no additional AEs or SAEs. Thus, sintilimab plus bevacizumab may be a better first line choice for the treatment of patients with unresectable HCC.

**Systematic Review Registration:**

PROSPEROI [https://www.crd.york.ac.uk/PROSPERO/index.php], identifier CRD42021269734.

## Introduction

Hepatocellular carcinoma (HCC) is the fifth most common malignant tumor and the third most fatal tumor worldwide (1). It is extremely harmful to the health and life of patients and has become a serious social and public health problem. HCC is closely related to viral hepatitis, alcoholic liver disease, non-alcoholic fatty liver disease, cirrhosis, family history, genetic factors, and certain chemicals or drugs. Viral hepatitis is one of the most important factors involved in primary liver cancer (2). Technological advances and improved medical standards have increased the number of available treatment options for HCC, such as surgical treatment (hepatectomy and liver transplantation), ablation treatment (radiofrequency ablation, microwave ablation, and absolute alcohol injection), interventional treatment (hepatic artery embolization chemotherapy), molecular targeted drug therapy, antiviral and biological therapy, radiotherapy, systemic chemotherapy, and traditional Chinese medicine (3). However, because the early symptoms and signs of HCC are not obvious, patients are often diagnosed in the intermediate or late stages of liver cancer, which severely limits the treatment of this disease (4). Therefore, it is urgent to find effective and safe treatments for patients with unresectable HCC.

Patients with unresectable HCC often require systemic treatment. In the past few decades, research has focused on developing treatments that can effectively improve the prognosis of HCC. The number of first-line systemic therapies approved for HCC patients has greatly increased in the past several years, and many drugs and their combinations have been evaluated (5, 6). From 2007 to 2018, sorafenib was the only approved targeted drug for patients with unresectable HCC (7, 8). Sorafenib is an oral multi-target receptor tyrosine kinase inhibitor (TKI) that blocks the proliferation of tumor cells and inhibits the formation of new blood vessels by suppressing the RAF/MEK/ERK pathway and inhibiting vascular endothelial growth factor receptor 2/3 (VEGFR-2/3), platelet-derived growth factor receptors (PDGFR), and stem cell factor receptors (9, 10). The results of the SHARP phase III clinical trial showed that sorafenib was associated with a survival benefit compared with placebo (11). In the ten years after sorafenib was approved, many trials of systemic treatments for unresectable HCC were unsuccessful until the development of lenvatinib. The success of the phase III REFLECT trial led to the approval of lenvatinib for the first-line treatment of unresectable HCC (12). In this study, lenvatinib showed a non-inferior overall survival (OS) in the treatment of unresectable HCC compared with sorafenib. Since the approval of lenvatinib, other TKIs or immunotherapeutic drugs have gradually been approved as first-line or second-line treatment for HCC. In the phase III IMbrave 150 trial (13), the combination of atezolizumab plus bevacizumab was more effective than sorafenib regarding OS and progression-free survival (PFS). Recently, Ren et al. (14) published the results of phase III clinical trials showing that sintilimab plus bevacizumab was associated with better OS and PFS than sorafenib.

The choice of treatment strategy depends on liver function, tumor stage, and the patient’s clinical performance. However, there are still many uncertainties in the comparative efficacy of different therapies. A recent review of international guidelines for HCC indicated that despite similarities in treatment allocation recommendations, some discrepancies exist (15). In addition, some recommendations lack reliable or high-level evidence. Traditional meta-analyses are limited by the comparison of head-to-head treatments in the included studies. Therefore, the relative benefits of two therapies cannot be measured because they have never been directly compared in studies. Gaps in existing evidence affect real world treatment decisions, whereas network meta-analyses can integrate direct and indirect comparisons to provide estimates for the relative efficacy of many treatments.

The increase in first-line treatment options for unresectable HCC represents a major advancement in the management of this malignancy. However, further data analysis will be helpful for the selection of clinical treatments. In this study, we analyzed the recent literature and performed a network meta-analysis to systematically review and compare the OS and PFS outcomes of randomized trials examining first-line systemic treatment strategies for unresectable HCC. In addition, we analyzed data on probability of adverse events (AEs), which is important for treatment decisions to improve patient prognosis and quality of life.

## Materials and Methods

### Protocol and Registration

This network meta-analysis was registered with the PROSPEROI (CRD42021269734). Besides, the study was performed according to the Preferred Reporting Items for Systematic Reviews and Meta-Analyses (PRISMA) guidelines.

### Search Strategy and Literature Inclusion Criteria

The search strategy included the following terms: (“unresectable hepatocellular carcinoma” or “advanced hepatocellular carcinoma” or “metastatic hepatocellular carcinoma”) and (“first line systemic therapy” or “clinical trials” or “targeted agents” or “immune therapy”). The search terms were applied to Pubmed, Science Direct, Web of Science, Scopus, Ovid MEDLINE, Embase, Google Scholar, the Cochrane Library, EMbase, CNKI, CBM, VIP, and the Wanfang databases as well as the Cochrane Central Register of Controlled Trails to identify randomized controlled trials evaluating the efficacy of first-line chemotherapy, targeted treatment, and immunotherapy for HCC. The deadline was August 1, 2021. The corresponding reference documents and conference paper abstracts were searched manually. Titles, abstracts, and full texts were screened to identify eligible studies.

The inclusion criteria were as follows: (1) research objective: history of HCC; (2) randomized-controlled studies with head-to-head comparisons of at least two treatment arms, and similar articles published by the same author recently; (3) systemic first-line therapy for unresectable or metastatic HCC; (4) outcome indicators were OS, PFS, and AEs, or a Kaplan-Meier curve that could be obtained from the original article or possibility of contacting the original author. If PFS was not reported, time to progression (TTP) was used instead. If the report lacked detailed information, or the data had already been reported (same institution, repeated period of patient recruitment), the study was excluded. Reviews, editorials, abstracts, letters, case reports, and expert opinions were excluded from the network meta-analysis.

### Data Screening and Quality Evaluation

Two researchers independently read the titles and abstracts of the retrieved documents and selected them according to the inclusion and exclusion criteria. Next, the full texts of the documents that may meet the inclusion criteria were read in detail, and finally the documents eligible for the meta-analysis were selected. Information was extracted using a single form that included the following items: basic conditions of the trial, baseline levels of patients in each group, intervention measures, important outcome indicators, and research quality evaluation. The quality evaluation was performed according to the quality criteria of the Cochrane Collaboration’s tool (15), and the relevant evaluation items were as follows: 1) whether a random sequence is generated; 2) whether the randomization is hidden; 3) whether the researchers and the subjects are double blinded; 4) whether the results are a blind evaluation; 5) whether the data result is complete; 6) whether there is any publication bias; 7) presence of other biases. When two researchers failed to reach an agreement, a third researcher was consulted until consensus was reached. Then the Grading of Recommendations, Assessment, Development and Evaluations (GRADE) approach was performed to assess quality of comparisons of treatments. Each outcome as high, moderate, low or very low using the GRADE rating system (16). In the GRADE system, randomized clinical trials begin as high quality evidence, but may be rated down by one or more of five categories of limitations: risk of bias, precision, consistency, directness and publication bias (17).

### Statistical Analysis

R version 3.6.1 (https://www.r-project.org), STATA 15.0 software (STATA, University of Texas Station, USA), and Excel 2010 (Excel, Microsoft Corp, USA) were used for statistical analyses of relevant data. Gemtc package v0.8-2 was used to perform Bayesian analysis. For OS, PFS and TTP, hazard ratio (HR) was utilized for comparisons. For AEs and SAEs, odds ratio (OR) was used for comparisons. The adjusted indirect comparison was calculated using Bayesian methods embedded in the following formula: ln(HR)= [ln(UL-HR)+ln(LL-HR)]/2; seln(HR)= [ln(UL-HR)-ln(LL-HR)]/(1.96×2); OR was calculated as follows; log(OR)=[log(UL-OR)+log(LL-OR)]/2; selog(OR)=[log(UL-OR)-log(LL-OR)]/(1.96×2). A consistency analysis of direct and indirect comparisons was performed. The ggplot2 package in R was used to perform the surface under the cumulative ranking curve (SUCRA) to discuss rank probability. P score, a frequentist analog to the SUCRA, was used to rank treatments (18). The I^2^ was calculated to assess the overall heterogeneity of the model. If no obvious heterogeneity was found (I^2^ <50% or P <0.1), the fixed effects model was adopted, otherwise the random effects model was adopted. The network meta-analysis was performed to synthesize the information of the included studies, and direct and indirect comparisons were made using methods based on the frequency school of Rücker et al. (18). A Funnel plot and Egger’s test were used to assess publication bias. A symmetrical graph indicated no obvious publication bias, whereas an asymmetrical graph indicated that there may be publication bias. For all calculations, a two-tailed p value <0.05 was considered statistically significant.

## Results

### Literature Search and Screening Results

**Figure 1:**
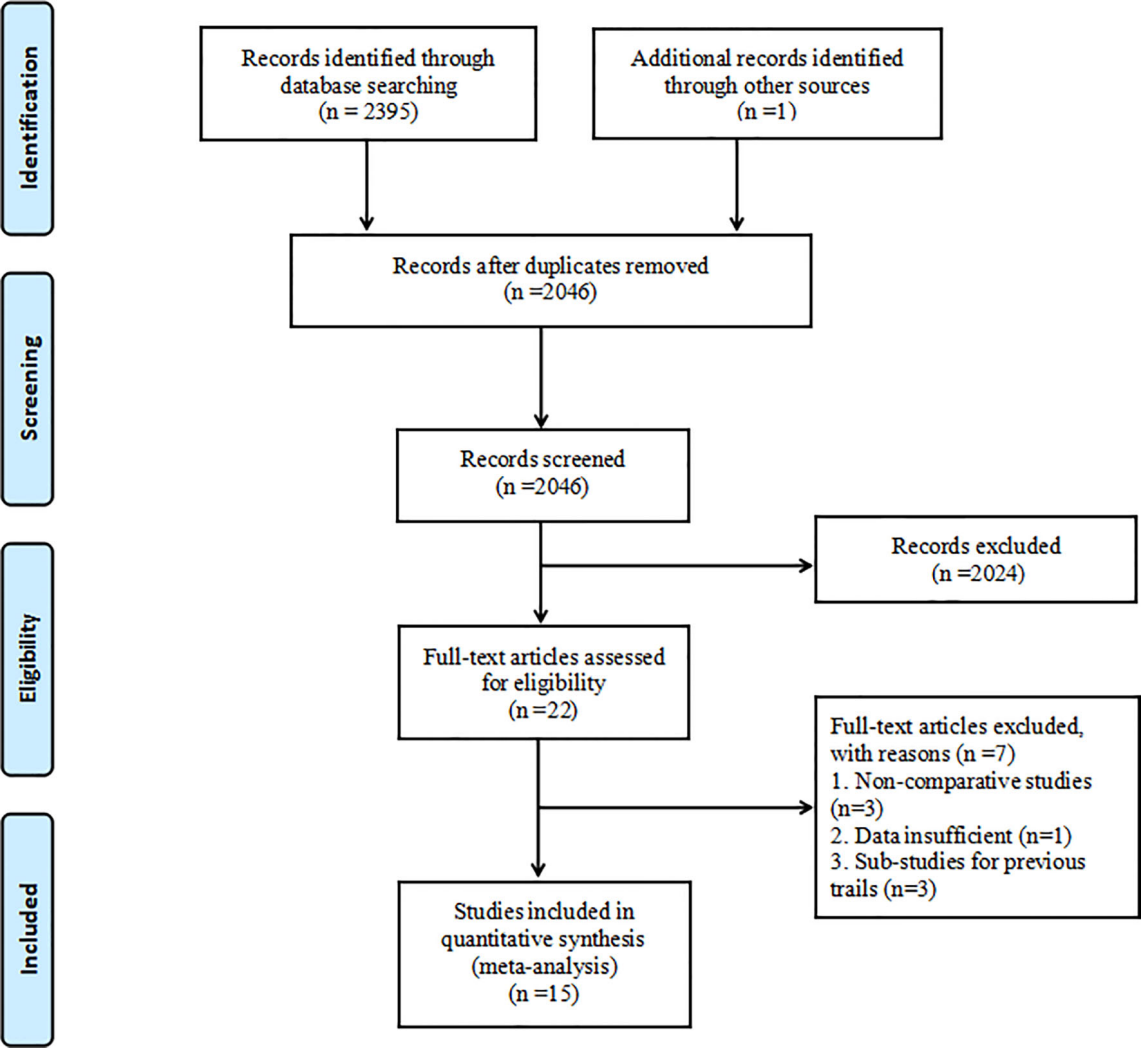
Flowchart of the study selection process.

The initial literature search retrieved 2396 articles. After deleting duplicate publications, 2046 articles remained. After screening titles and abstracts, 2024 articles were excluded. Full-text review resulted in the removal of seven articles. Ultimately, 15 studies including 9005 patents were included in the network meta-analysis (11–14, 19–29). The literature selection process is described in **Figure 1**.

### Study Characteristics and Quality Assessment

The 15 clinical trials were all double-arm randomized controlled studies, and the number of patients ranged from 83 to 1155. The number of men in each study was greater than the number of women. There were 80% of studies performed in a multinational environment. Two studies used mRECIST, five studies used RECIST1.0, and eight studies used RECIST 1.1. There were ten phase III studies, three phase II studies, and two phase II–III studies. Additional details are shown in **Table 1**. There was no evidence of serious imbalance in the distribution of effect correction factors in the entire network experiment. The connected network diagram including all the evidence is shown in **Supplementary Figure 1**. The same drug was used at the same dosage in all studies. Examination of patients’ baseline characteristics, treatment stage, and treatment plan showed no evidence that the transitivity hypothesis was violated in any network.

**Table 1:** Characteristics of the included trials.

Author	Year	Trial	RCT phase	Treatment Arm	Comparative Arm	Patients number	Region	Male	Mean/median age	HBV	HCV	Child A	BCLC C	PVI	EHS	Outcome
Llove (11)	2008	SHARP	III	Sorafenib	Placebo	602	US Eu AusNZ	87.0%	65.6	18.4%	28.1%	96.5%	82.4%	38.4%	51.3%	OS, PFS, AEs
Cheng (19)	2009	NCT00492752	III	Sorafenib	Placebo	226	AP	85.4%	52.5	73%	8.4%	97.3%	95.6%	35.4%	68.6%	OS, PFS, AEs
Cheng (20)	2013	SUN1170	III	Sunitinib	Sorafenib	1074	AP	83.3%	55.1	53.8%	21.6%	99.6%	85.3%	32.1%	–	OS, PFS
Johnson (21)	2013	BRISK-FL	III	**Brivanib**	Sorafenib	1155	US Eu AP AusNZ	83.7%	57.8	44.3	20.3%	92.0%	77.3%	19.3%	49.7	OS, PFS, AEs
Cheng (22)	2015	NCT01033240	II	Tigatuzumab plus Sorafenib	Sorafenib	162	AP US	82.7%	63	50%	33.9%	100.0%	–	–	–	OS, PFS, AEs
Cainap (23)	2015	NCT01009593	III	Linifanib	Sorafenib	1035	US Eu AP AusNZ	85%	56.6	53.2%	25%	94.4%	82.2%	43.4%	58.3%	OS, PFS, AEs
Zhu (24)	2015	SEARCH	III	Erlotinib plus Sorafenib	Sorafenib	720	US Eu AP	80.7%	–	35.4%	26.5%	97.4%	85%	40.4%	58.9%	OS, PFS, AEs
Kudo (25)	2017	NCT02400788	II	Resminostat plus Sorafenib	Sorafenib	164	AP	–	–	–	–	–	–	–	–	OS
Kudo (12)	2018	REFLECT	III	Lenvatinib	Sorafenib	954	US Eu AP	84.0%	58.0	50%	23.0%	99.0%	79.0%	21.0%	61.0%	OS, PFS, AEs
Abou-Alfa (26)	2019	CALGB80802	III	Sorafenib plus doxorubicin	Sorafenib	356	AP US Canada	67.1%	62	9.3%	19.7%	100.0%	–	–	–	OS, PFS
Yau (27)	2019	CheckMate 459/III	III	Nivolumab	Sorafenib	743	AP, Eu, US, Canada	85%	65	–	–	–	–	–	–	OS
Assenat (28)	2019	NCT00941967	II	Sorafenib	Sorafenib plus GEMOX	83	Eu	89.2%	62	3.6%	15.7%	–	85.5%	26.5%	68.7%	PFS, AEs
Qin (29)	2020	NCT02645981	II–III	Donafenib	Sorafenib	659	AP	86.8%	53	90.1%	1.8%	97.4%	87.4%	73.4%	31.4%	OS, PFS, AEs
Finn (13)	2020	IMbrave150	III	Atezolizumab plus Bevacizumab	Sorafenib	501	AP, US, Australia, Eu	82.6%	64	47.9%	8.6%	100.0%	81.6%	39.9%	60.9%	OS, PFS, AEs
Ren (14)	2021	NCT03794440	II–III	Sintilimab plus bevacizumab	Sorafenib	571	AP	88.4%	53	94.2%	2.5%	95.8%	85.5%	27.1%	74.1%	OS, PFS, AEs

US, the United State; Eu, Europe; AP, Asia-Pacific; HBV, hepatitis Bvirus; HCV, hepatitis C virus; BCLC, Barcelona Clinic Liver Cancer; PVI, portal vein invasion; EHS, extrahepatic spread; OS, overall survival; PFS, progression-free survival; AEs, adverse events.

Brivanib, a small-molecule tyrosine kinase inhibitor, is the first oral selective dual inhibitor of fibroblast growth factors (FGF) and vascular endothelial growth factor (VEGF) signaling.

The results of the quality criteria of the Cochrane Collaboration’s tool showed that only three studies did not mention random methods, and nine trials were open-label design. The included studies were found to have a low risk of bias across all six domains. In general, the quality of the clinical trials included in the study was relatively high. The quality of each included article was detailed in **Supplementary Figure 2**. The certainty of the evidence for the each pairwise treatment comparison was overall moderate (due to imprecision) to high (**Supplementary Tables 1-4**).

### Overall Survival

**Figure 2:**
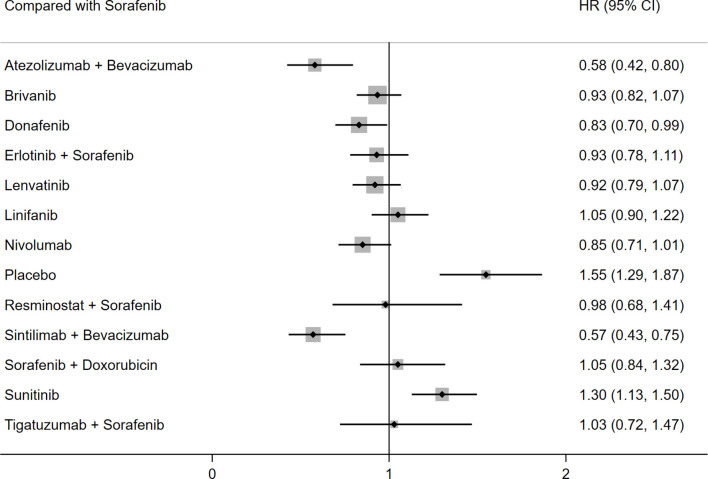
Forest plot for HR of OS.

OS was reported in 14 studies including 14 different interventions. There was no significant heterogeneity between studies (I^2^ = 6%), and the fixed effects model was adopted. P score for OS showed that the best OS outcomes were obtained with sintilimab plus bevacizumab over placebo (HR: 0.36; 95% CI: 0.25–0.52; P score: 0.891); atezolizumab plus bevacizumab ranked second (HR: 0.37; 95% CI: 0.26–0.54; P score: 0.880), and donafenib ranked third (HR: 0.54; 95% CI: 0.41–0.69; P score: 0.652). Compared with sorafenib, sintilimab plus bevacizumab (HR: 0.57; 95% CI: 0.43–0.75), atezolizumab plus bevacizumab (HR: 0.58; 95% CI: 0.58–0.80), and donafenib (HR: 0.83; 95%CI: 0.70–0.99) were associated with better OS. However, sorafenib showed better results than placebo and sunitinib. There was no significant difference between the efficacy of the remaining treatment strategies and sorafenib (**Table 2** and **Figure 2**). P score for the outcome of OS were shown in **Supplementary Figure 3A**.

**Table 2:** Network meta-analyses for OS.

Atezolizumab + Bevacizumab													
0.62 (0.44, 0.88)	Brivanib												
0.70 (0.49, 1.00)	1.13 (0.90, 1.4)	Donafenib											
0.62 (0.43, 0.90)	1.00 (0.80, 1.25)	0.89 (0.7, 1.14)	Erlotinib + Sorafenib										
0.63 (0.44, 0.89)	1.02 (0.83, 1.24)	0.90 (0.72, 1.13)	1.01 (0.80, 1.27)	Lenvatinib									
0.55 (0.39, 0.79)	0.89 (0.73, 1.09)	0.79 (0.63, 1.00)	0.89 (0.70, 1.12)	0.88 (0.71, 1.08)	Linifanib								
0.68 (0.48, 0.98)	1.10 (0.88, 1.37)	0.98 (0.76, 1.25)	1.09 (0.85, 1.40)	1.08 (0.86, 1.36)	1.24 (0.98, 1.56)	Nivolumab							
0.37 (0.26, 0.54)	0.60 (0.48, 0.76)	0.54 (0.41, 0.69)	0.60 (0.46, 0.78)	0.59 (0.47, 0.75)	0.68 (0.53, 0.86)	0.55 (0.43, 0.71)	Placebo						
0.59 (0.37, 0.96)	0.95 (0.65, 1.41)	0.85 (0.56, 1.27)	0.95 (0.63, 1.42)	0.94 (0.63, 1.39)	1.07 (0.72, 1.59)	0.87 (0.58, 1.30)	1.58 (1.05, 2.38)	Resminostat + Sorafenib					
1.02 (0.67, 1.55)	1.64 (1.20, 2.23)	1.46 (1.05, 2.02)	1.63 (1.17, 2.27)	1.61 (1.18, 2.21)	1.84 (1.34, 2.53)	1.49 (1.07, 2.07)	2.77 (1.94, 3.78)	1.72 (1.09, 2.72)	Sintilimab +Bevacizumab				
0.58 (0.42, 0.80)	0.93 (0.82, 1.07)	0.83 (0.70, 0.99)	0.93 (0.78, 1.11)	0.92 (0.79, 1.07)	1.05 (0.90, 1.22)	0.85 (0.71, 1.01)	1.55 (1.29, 1.87)	0.98 (0.68, 1.41)	0.57 (0.43, 0.75)	Sorafenib			
0.55 (0.37, 0.82)	0.89 (0.68, 1.16)	0.79 (0.59, 1.05)	0.89 (0.66, 1.18)	0.88 (0.67, 1.15)	1.00 (0.76, 1.32)	0.81 (0.61, 1.08)	1.47 (1.10, 1.98)	0.93 (0.61, 1.43)	0.54 (0.38, 0.78)	0.95 (0.76, 1.20)	Sorafenib +Doxorubicin		
0.45 (0.32, 0.63)	0.72 (0.59, 0.87)	0.64 (0.51, 0.80)	0.72 (0.57, 0.90)	0.71 (0.58, 0.87)	0.81 (0.66, 0.99)	0.65 (0.52, 0.82)	1.19 (0.94, 1.50)	0.75 (0.51, 1.12)	0.44 (0.32, 0.60)	0.77 (0.67, 0.89)	0.81 (0.62, 1.06)	Sunitinib	
0.56 (0.35, 0.90)	0.91 (0.62, 1.32)	0.81 (0.54, 1.20)	0.90 (0.61, 1.34)	0.89 (0.61, 1.31)	1.02 (0.69, 1.50)	0.83 (0.56, 1.22)	1.50 (1.01, 2.24)	0.95 (0.57, 1.58)	0.55 (0.35, 0.87)	0.97 (0.68, 1.38)	1.02 (0.67, 1.56)	1.26 (0.86, 1.85)	Tigatuzumab + Sorafenib

OS, overall survival.

### Progression Free Survival

**Figure 3:**
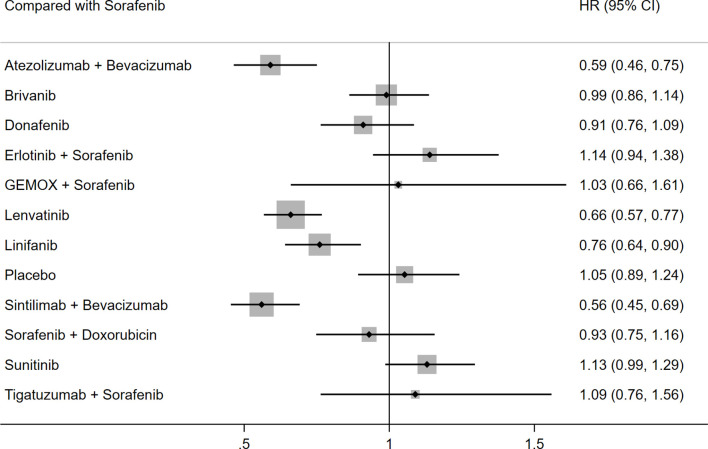
Forest plot for HR of PFS.

PFS was reported in 13 studies including 13 different interventions. No significant heterogeneity was found between the studies (I^2^ = 10%), and the fixed effects model was used. The results of SUCRA and P score showed that PFS was best in the sintilimab plus bevacizumab group over placebo (HR: 0.53; 95% CI: 0.41–0.70; P score: 0.845), atezolizumab plus bevacizumab ranked second (HR: 0.56; 95% CI: 0.42–0.75; P score: 0.818), and lenvatinib ranked third (HR: 0.63; 95% CI: 0.50–0.78; P score: 0.754). Detailed P scores for the outcome of PFS were shown in **Supplementary Figure 3B**. Compared with sorafenib, sintilimab plus bevacizumab (HR: 0.56; 95% CI: 0.45–0.69), atezolizumab plus bevacizumab (HR: 0.59; 95%CI: 0.46-0.75), lenvatinib (HR: 0.66; 95% CI: 0.57–0.77), and linifanib (HR: 0.76; 95% CI: 0.64–0.90) were associated with better PFS. There was no significant difference between the remaining treatment strategies and sorafenib regarding PFS (**Table 3** and **Figure 3**).

**Table 3:** Network meta-analyses for PFS.

Atezolizumab + Bevacizumab												
0.60 (0.45, 0.79)	Brivanib											
0.65 (0.48, 0.87)	1.09 (0.87, 1.36)	Donafenib										
0.52 (0.38, 0.70)	0.87 (0.69, 1.10)	0.80 (0.62, 1.03)	Erlotinib + Sorafenib									
0.57 (0.35, 0.95)	0.96 (0.60, 1.53)	0.88 (0.55, 1.42)	1.10 (0.68, 1.79)	GEMOX + Sorafenib								
0.89 (0.67, 1.19)	1.50 (1.22, 1.84)	1.38 (1.09, 1.74)	1.73 (1.36, 2.20)	1.56 (0.98, 2.49)	Lenvatinib							
0.78 (0.58, 1.04)	1.30 (1.05, 1.62)	1.20 (0.94, 1.53)	1.50 (1.16, 1.93)	1.36 (0.84, 2.18)	0.87 (0.69, 1.09)	Linifanib						
0.56 (0.42, 0.75)	0.94 (0.76, 1.17)	0.87 (0.68, 1.10)	1.08 (0.84, 1.39)	0.98 (0.61, 1.57)	0.63 (0.50, 0.78)	0.72 (0.57, 0.92)	Placebo					
1.05 (0.77, 1.45)	1.77 (1.37, 2.27)	1.63 (1.23, 2.14)	2.04 (1.53, 2.70)	1.84 (1.13, 3.00)	1.18 (0.91, 1.53)	1.36 (1.04, 1.78)	1.88 (1.44, 2.45)	Sintilimab + Bevacizumab				
0.59 (0.46, 0.75)	0.99 (0.86, 1.14)	0.91 (0.76, 1.09)	1.14 (0.94, 1.38)	1.03 (0.66, 1.61)	0.66 (0.57, 0.77)	0.76 (0.64, 0.90)	1.05 (0.89, 1.24)	0.56 (0.45, 0.69)	Sorafenib			
0.63 (0.46, 0.88)	1.06 (0.82, 1.38)	0.98 (0.74, 1.29)	1.23 (0.92, 1.64)	1.11 (0.68, 1.82)	0.71 (0.54, 0.92)	0.82 (0.62, 1.08)	1.13 (0.86, 1.49)	0.60 (0.45, 0.82)	1.08 (0.86, 1.34)	Sorafenib + Doxorubicin		
0.52 (0.40, 0.69)	0.88 (0.72, 1.06)	0.81 (0.64, 1.01)	1.01 (0.80, 1.27)	0.91 (0.57, 1.45)	0.58 (0.48, 0.72)	0.67 (0.54, 0.84)	0.93 (0.75, 1.15)	0.50 (0.39, 0.64)	0.88 (0.77, 1.01)	0.82 (0.64, 1.06)	Sunitinib	
0.54 (0.35, 0.83)	0.91 (0.62, 1.33)	0.84 (0.56, 1.24)	1.05 (0.70, 1.57)	0.95 (0.54, 1.67)	0.61 (0.41, 0.89)	0.70 (0.47, 1.04)	0.96 (0.65, 1.43)	0.51 (0.34, 0.78)	0.92 (0.64, 1.31)	0.85 (0.56, 1.30)	1.04 (0.71, 1.52)	Tigatuzumab + Sorafenib

PFS, progression-free survival.

### Safety

**Figure 4:**
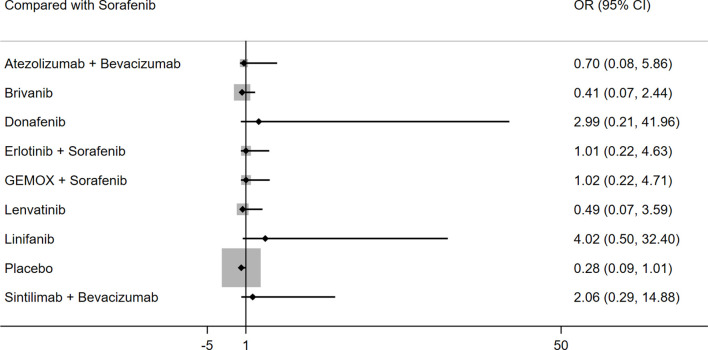
Forest plot for OR of any grade AEs.

Ten studies including ten different interventions reported on any grade AEs. There was no significant heterogeneity between the studies (I^2^ = 5%), and the fixed effects model was adopted. The results of the indirect comparison of the different interventions are shown in **Table 4**. Linifanib was the only drug showing a higher likelihood of any grade AEs than placebo, whereas the other treatments did not show statistically significant differences (**Table 4** and **Figure 4**). The results of ranking treatment showed that linifanib ranked 1/10 for any grade AEs (P score: 0.852), donafenib ranked 2/10 (P score: 0.763), sintilimab plus bevacizumab ranked 3/10 (P score: 0.724), sorafenib ranked 6/10 (P score: 0.523) and atezolizumab plus bevacizumab ranked 7/10 (P score: 0.402) (higher ranking indicated a high incidence of AEs, **Supplementary Figure 3C**).

**Table 4:** Network meta-analyses for any grade AEs.

Atezolizumab + Bevacizumab									
1.69 (0.11, 26.75)	Brivanib								
0.23 (0.01, 7.02)	0.14 (0.01, 3.37)	Donafenib							
0.69 (0.05, 9.05)	0.41 (0.04, 4.23)	2.96 (0.14, 59.14)	Erlotinib + Sorafenib						
0.68 (0.05, 9.12)	0.40 (0.04, 4.15)	2.92 (0.14, 58.84)	0.99 (0.11, 8.51)	GEMOX + Sorafenib					
1.42 (0.08, 25.51)	0.83 (0.06, 12.03)	6.01 (0.22, 164.01)	2.04 (0.17, 24.01)	2.07 (0.17, 24.36)	Lenvatinib				
0.17 (0.01, 3.50)	0.10 (0.01, 1.56)	0.75 (0.03, 21.89)	0.25 (0.02, 3.22)	0.25 (0.02, 3.28)	0.12 (0.01, 2.18)	Linifanib			
2.45 (0.21, 26.97)	1.44 (0.16, 11.84)	10.38 (0.55, 183.07)	3.59 (0.48, 23.72)	3.64 (0.49, 23.92)	1.73 (0.16, 16.98)	14.02 (1.23, 151.60)	Placebo		
0.34 (0.02, 6.17)	0.20 (0.01, 2.79)	1.43 (0.05, 40.65)	0.49 (0.04, 5.69)	0.50 (0.04, 5.87)	0.24 (0.01, 3.93)	1.94 (0.11, 34.50)	0.14 (0.01, 1.46)	Sintilimab + Bevacizumab	
0.70 (0.08, 5.86)	0.41 (0.07, 2.44)	2.99 (0.21, 41.96)	1.01 (0.22, 4.63)	1.02 (0.22, 4.71)	0.49 (0.07, 3.59)	4.02 (0.50, 32.40)	0.28 (0.09, 1.01)	2.06 (0.29, 14.88)	Sorafenib

AEs, adverse events.

**Figure 5:**
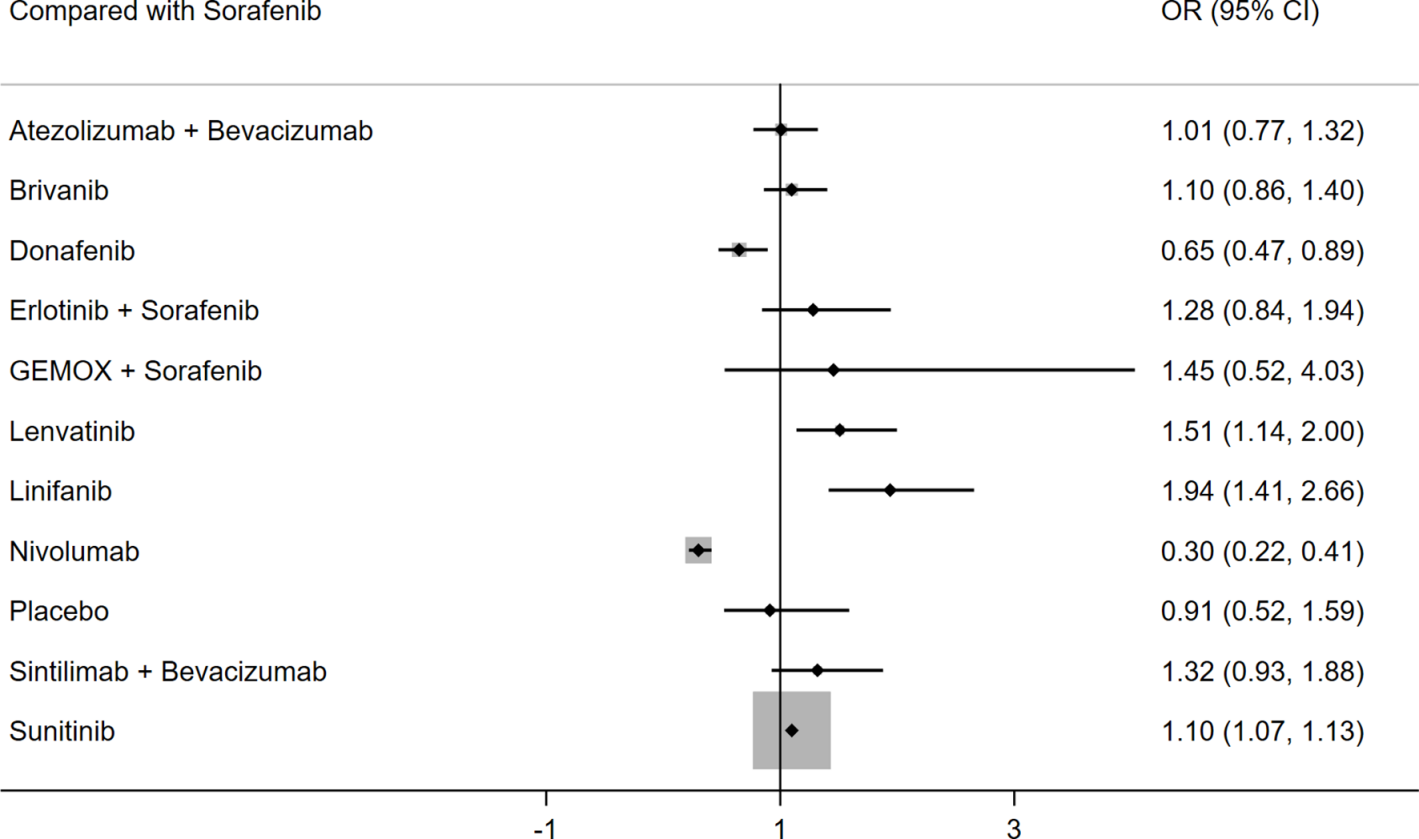
Forest plot for OR of grade 3-5 AEs.

Eleven studies reported on grade 3–5 AEs. The fixed effects model was used because I^2^ was 8%. **Table 5** shows the results of the direct and indirect comparisons of different interventions. Nivolumab (HR: 0.30; 95% CI: 0.22–0.41) and donafenib (HR: 0.65; 95% CI: 0.47–0.89) showed a lower probability of grade 3–5 AEs than sorafenib, whereas lenvatinib (HR: 1.51; 95% CI: 1.14–2.00) and linifanib (HR: 1.94; 95% CI: 1.41–2.66) showed a higher probability of grade 3–5 AEs than sorafenib (**Figure 5**). The results of SUCRA for grade 3-5 AEs demonstrated that linifanib ranked 1/12 (P score: 0.778), lenvatinib ranked 2/12 (P score: 0.676), sintilimab plus bevacizumab ranked 4/12 (P score: 0.609), and atezolizumab plus bevacizumab ranked 8/12 (P score: 0.465) (**Supplementary Figure 3D**). Despite their association with favorable OS and PFS, sintilimab plus bevacizumab or atezolizumab plus bevacizumab had no additional side effects compared with other therapies.

**Table 5:** Network meta-analyses for grade 3-5 AEs.

Atezolizumab + Bevacizumab											
0.92 (0.64, 1.32)	Brivanib										
1.55 (1.02, 2.36)	1.69 (1.13, 2.52)	Donafenib									
0.79 (0.48, 1.29)	0.86 (0.53, 1.39)	0.51 (0.30, 0.86)	Erlotinib + Sorafenib								
0.69 (0.24, 1.98)	0.75 (0.27, 2.15)	0.45 (0.15, 1.30)	0.88 (0.29, 2.65)	GEMOX + Sorafenib							
0.67 (0.45, 0.98)	0.73 (0.50, 1.06)	0.43 (0.28, 0.66)	0.85 (0.51, 1.40)	0.96 (0.34, 2.78)	Lenvatinib						
0.52 (0.34, 0.79)	0.57 (0.38, 0.84)	0.33 (0.21, 0.52)	0.66 (0.39, 1.11)	0.75 (0.26, 2.18)	0.78 (0.51, 1.19)	Linifanib					
3.35 (2.21, 5.07)	3.65 (2.45, 5.44)	2.16 (1.38, 3.38)	4.26 (2.52, 7.19)	4.84 (1.66, 14.09)	5.01 (3.28, 7.66)	6.44 (4.11, 10.08)	Nivolumab				
1.11 (0.60, 2.06)	1.21 (0.66, 2.22)	0.71 (0.37, 1.35)	1.41 (0.70, 2.84)	1.60 (0.50, 5.09)	1.66 (0.89, 3.09)	2.13 (1.12, 4.04)	0.33 (0.17, 0.63)	Placebo			
0.76 (0.49, 1.19)	0.83 (0.54, 1.27)	0.49 (0.31, 0.79)	0.97 (0.56, 1.68)	1.10 (0.38, 3.25)	1.14 (0.73, 1.80)	1.47 (0.92, 2.35)	0.23 (0.14, 0.37)	0.69 (0.36, 1.34)	Sintilimab + Bevacizumab		
1.01 (0.77, 1.32)	1.10 (0.86, 1.40)	0.65 (0.47, 0.89)	1.28 (0.84, 1.94)	1.45 (0.52, 4.03)	1.51 (1.14, 2.00)	1.94 (1.41, 2.66)	0.30 (0.22, 0.41)	0.91 (0.52, 1.59)	1.32 (0.93, 1.88)	Sorafenib	
0.92 (0.70, 1.20)	1.00 (0.78, 1.28)	0.59 (0.43, 0.81)	1.17 (0.77, 1.77)	1.32 (0.48, 3.67)	1.37 (1.03, 1.82)	1.76 (1.28, 2.42)	0.27 (0.2, 0.38)	0.83 (0.47, 1.45)	1.2 (0.84, 1.71)	0.91 (0.89, 0.93)	Sunitinib

AEs, adverse events.

### Assessment of Publication Bias

The funnel plot and Egger’s test revealed no significant bias across studies in the first-line treatment (**Supplementary Figure 4**).

## Discussion

First-line systemic treatment options for unresectable HCC have increased in the past decade, and several drugs such as sorafenib, lenvatinib, and atezolizumab plus bevacizumab, as well as nivolumab and systemic chemotherapy are currently recommended for selected patients (30–32). However, because most clinical trials of patients with unresectable HCC are double-armed, it is difficult for oncologists to compare existing treatment options. Therefore, we performed a comprehensive and systematic review and network meta-analysis of first-line systemic treatments for unresectable HCC to compare the therapeutic efficacy and safety of various treatments and facilitate treatment selection for clinicians.

In this network meta-analysis, we compared the efficacy and safety of targeted drugs and immunotherapy strategies approved as first-line systemic treatment for unresectable HCC. The results showed that sintilimab plus bevacizumab had the best OS outcomes; atezolizumab plus bevacizumab ranked second, and donafenib ranked third. The top three treatments associated with better PFS were sintilimab plus bevacizumab, atezolizumab plus bevacizumab, and lenvatinib. As for intervention ranking by P value, sintilimab plus bevacizumab ranked first for OS and PFS over atezolizumab plus bevacizumab. Besides, there were no statistically significant differences in any grade AEs except in patients treated with linifanib, which was associated with a higher probability of any grade AEs than placebo. Donafenib showed a lower probability, whereas lenvatinib and linifanib showed a higher probability of SAEs than sorafenib. So it can be concluded that sintilimab plus bevacizumab was associated with the most favorable OS and PFS with no additional AEs or SAEs.

Many drugs for unresectable HCC were approved in the past decade. Sorafenib was the first approved molecular targeted drug that improved the prognosis of patients with unresectable HCC. Sorafenib is a multi-target tyrosine kinase inhibitor that can not only inhibit VEGFR, fibroblast growth factor receptor (FGFR), and PDGFR, but also RAS/RAF/ERK signaling and the proto-oncogene c-KIT. It thus has the ability to block the formation of tumor blood vessels and inhibit the proliferation of liver cancer cells (33). The therapeutic efficacy of sorafenib was reported previously; it was shown to increase the OS of Western HCC patients from 10.7 months in 2008 to 15.1 months in 2013, and in Asian HCC patients from 6.5 months to 11 months (34). It is worth mentioning regorafenib, another TKI inhibitor, is the first and only second-line treatment that can significantly improve the OS of HCC patients. It has been reported that regorafenib can prolong the OS and PFS of HCC patients who are resistant to sorafenib (35). In addition, a meta-analysis demonstrated that regorafenib is an effective and safe treatment in HCC patients who progress on sorafenib (36).

Lenvatinib is a type of TKI that can inhibit the activity of VEGF, RET, FGFR-4, PDGFR, RET, and KIT, and weaken the formation of tumor blood vessels and the proliferation of tumor cells (37). Lenvatinib has been used for the treatment of solid tumors such as thyroid cancer, kidney cancer, and HCC (38, 39). Kudo et al. (12) found no statistically significant difference in OS between the lenvatinib group and the sorafenib group, although lenvatinib was superior to sorafenib regarding the objective response rate (ORR). This is consistent with the results of the current network meta-analysis.

The occurrence and progression of HCC are closely related to the basic inflammatory state of the liver, such as viral hepatitis B and C and alcoholic cirrhosis. In this inflammatory state, various immune-related cells and cytokines in the liver form an immunosuppressive microenvironment for HCC. There are three mechanisms by which liver cancer cells achieve immune tolerance and grow in this environment. First, effector T cells such as CD8+ T cells and natural killer cells cannot break through the immunosuppressive microenvironment. Second, effector T cells can break through the immunosuppressive microenvironment, but they cannot recognize tumor cells. Third, after effector T cells break through the immunosuppressive microenvironment and recognize tumor cells, they are inactivated or transformed into immunosuppressive cells (40, 41). Although the specific mechanism remains to be elucidated, antitumor effects can theoretically be induced by changing the functional state, number, and surface molecules of immune cells in the immunosuppressive microenvironment. In fact, immunotherapy drugs that target the tumor microenvironment or regulate immune cell homeostasis have shown clinical benefits in several solid tumors, such as lung cancer, kidney cancer, and melanoma (42–44). Most of these immunotherapy drugs exert anti-tumor effects by inhibiting the immune checkpoint programmed death factor 1 (PD-1)/programmed death factor ligand 1 (PD-L1) pathway. In HCC, CD8+ T cells express PD1 receptors, whereas tumor cells and peritumoral cells express their ligand PD-L1, which inactivates CD8+ T cells and leads to the occurrence of immune tolerance (45). Therefore, the use of immunotherapy to reshape the tumor microenvironment of HCC and reverse tumor immune tolerance is a promising strategy for the treatment of unresectable HCC.

Among the experimental immunotherapies for unresectable HCC, the first breakthrough was the immune checkpoint PD-1 inhibitor. Nivolumab is a human immunoglobulin G4 monoclonal antibody and also the first immune checkpoint inhibitor for HCC approved by the FDA. In the CheckMate040 study (46), the ORR of nivolumab for unresectable HCC was 15–20% and the disease control rate reached 58–64%. Furthermore, the curative effect was long-lasting. However, in the clinical trial of nivolumab versus sorafenib, there was no significant difference in OS and PFS (27). In this network meta-analysis, nivolumab did not have a significant therapeutic advantage over other therapies. In the IMbrave150 study (13), researchers used a PD-L1 monoclonal antibody combined with anti-angiogenesis targeted drugs to treat 336 patients with metastatic or unresectable HCC. They found that atezolizumab plus bevacizumab reduced the patient’s risk of death by 42% and increased the 12-month survival rate to 67.2%, which significantly prolonged the survival of patients. Thus, this combination was approved as first-line treatment for patients with unresectable HCC who did not receive systemic treatment. In another clinical trial (47), the 6-month OS of unresectable HCC patients in the camrelizumab plus apatinib treatment group was 66%, whereas the 12 months OS was 77%. However, this study was a single-arm uncontrolled trial, and it was thus not included in the present analysis. The combination of immunity and TKI anti-angiogenesis therapy has obvious effects, highlighting the characteristics and advantages of immunotherapy, and it has gradually changed the existing clinical standards and treatment patterns of liver cancer. CTLA-4 is a transmembrane receptor on T cells that plays an important role in the negative regulation of T cells. It is also considered a promising therapeutic target for HCC. Although tremelimumab did not improve the prognosis of HCC patients in studies, the combination of nivolumab and ipilimumab showed greater survival benefits and safety than nivolumab alone (48, 49). In addition, lymphocyte activation gene 3 (LAG-3), T cell immunoglobulin and ITIM domain (TIGIT), T cell immunoglobulin and mucin-containing domain-3 (TIM-3), and B and T lymphocyte attenuation factor (BTLA) are new immune checkpoints in HCC, and further research is needed to improve the prognosis of liver cancer patients (50).

The first-line systemic treatment strategies for unresectable HCC range from traditional TKI-targeted drugs to immune checkpoint inhibitor single drugs, and then to the exploration of immune checkpoint inhibitor and anti-angiogenesis combined therapy. These treatments have improved the survival outcomes of patients with unresectable HCC. The latest clinical trial demonstrated that sintilimab plus bevacizumab achieved favorable treatment effects (14). Sintilimab is a selective anti-PD-1 antibody that inhibits the interaction between PD-1 and its ligand. It has been approved for the treatment of relapsed or refractory classical Hodgkin’s lymphoma (51). The median PFS of the sintilimab plus bevacizumab group was significantly better than that of the sorafenib group (4.6 months vs. 2.8 months), and the risk of disease progression or death decreased by 44%. The OS of patients treated with the combination of sintilimab and bevacizumab was remarkably improved. Therefore, sintilimab plus bevacizumab resulted in significantly better survival than existing standard treatments, with significantly prolonged OS and PFS. Additionally, it was the first phase III trial to show that an anti-PD-1 antibody combined with an anti-VEGF antibody significantly improves the prognosis of patients with unresectable HCC. In the current network meta-analysis, sintilimab plus bevacizumab was associated with best OS and PFS and ranked first for both OS and PFS among those various treatment options. What’s more, there were no significant differences in any grade AEs and grade 3–5 AEs between sintilimab plus bevacizumab and other therapies.

The present network meta-analysis showed that sintilimab plus bevacizumab had better efficacy than atezolizumab plus bevacizumab. There are several possible confounding factors for this result. First, the survival outcome is affected by second-line treatment modalities in different regions. Patients treated with sintilimab plus bevacizumab were all from Asia, where local regional treatments such as traditional transcatheter arterial chemoembolization, hepatic artery infusion chemotherapy, or radiotherapy are used and combined with second-line systemic treatments in advanced HCC. The patients included in the IMbrave150 study were mainly from Western countries (59.9%), where the preferable second-line treatments are systemic. In addition, patients in the atezolizumab plus bevacizumab group were older than those in the sintilimab plus bevacizumab group, which would increase non-cancer specific death risks. Finally, the proportion of HBV-positive patients in the sintilimab plus bevacizumab group was higher than that in the atezolizumab plus bevacizumab group. Different background liver diseases can also affect prognosis. Additional studies are needed to evaluate the difference in efficacy between atezolizumab plus bevacizumab and sintilimab plus bevacizumab.

Although this study provided important information regarding the efficacy and safety of the latest first-line systemic treatment options for unresectable HCC, the study had several limitations. First, because direct evidence was more important than indirect evidence, the lack of direct evidence in first-line trials limited the power of this analysis. Second, not all included studies had a large sample size, which might undermine the statistical power of the network meta-analysis. Additionally, some of the qualified studies did not directly provide survival data, and the corresponding HR and 95% CI were extracted from the survival curve, which might lead to micro-statistical errors.

The current network meta-analysis demonstrated that sintilimab plus bevacizumab ranked first and was associated with better OS and PFS, so it might be the best choice in the treatment of unresectable HCC. There was no significant difference in AEs between sintilimab plus bevacizumab and other drugs. Thus, sintilimab plus bevacizumab is an effective treatment for unresectable HCC.

## Data Availability Statement

The original contributions presented in the study are included in the article/supplementary material. Further inquiries can be directed to the corresponding author.

## Author Contributions

WL and XY were responsible for designing the study. WL and BQ analyzed the data, and WL wrote the manuscript. SL, BT, ML and RC helped literature searching and data analysis. XY and ZR contributed to revising the manuscript. All authors contributed to the article and approved the submitted version.

## Funding

This research was supported by National Natural Science Foundation of China (No: 81972889) and Exploratory Clinical Research Projects of National Clinical Research Center for Interventional Medicine (2021-001).

## Conflict of Interest

The authors declare that the research was conducted in the absence of any commercial or financial relationships that could be construed as a potential conflict of interest.

## Publisher’s Note

All claims expressed in this article are solely those of the authors and do not necessarily represent those of their affiliated organizations, or those of the publisher, the editors and the reviewers. Any product that may be evaluated in this article, or claim that may be made by its manufacturer, is not guaranteed or endorsed by the publisher.

## References

[1] Kim J Min JH Kim SK Shin SY Lee MW . Detection of Hepatocellular Carcinoma in Contrast-Enhanced Magnetic Resonance Imaging Using Deep Iearning Classifier: A Multi-Center Retrospective Study. Sci Rep (2020) 10:9458. doi: 10.1038/s41598-020-65875-4 32527998 PMC7289813

[2] Liu Z Jiang Y Yuan H Fang Q Cai N Suo C . The Trends in Incidence of Primary Liver Cancer Caused by Specific Etiologies: Results From the Global Burden of Disease Study 2016 and Implications for Liver Cancer Prevention. J Hepatol (2019) 70:674–83. doi: 10.1016/j.jhep.2018.12.001 30543829

[3] Couri T Pillai A . Goals and Targets for Personalized Therapy for HCC. Hepatol Int (2019) 13:125–37. doi: 10.1007/s12072-018-9919-1 30600478

[4] Feng J Dai W Mao Y Wu L Li J Chen K . Simvastatin Re-Sensitizes Hepatocellular Carcinoma Cells to Sorafenib by Inhibiting HIF-1α/PPAR-γ/PKM2-Mediated Glycolysis. J Exp Clin Cancer Res (2020) 39:24. doi: 10.1186/s13046-020-1528-x 32000827 PMC6993409

[5] Kudo M . Recent Advances in Systemic Therapy for Hepatocellular Carcinoma in an Aging Society: 2020 Update. Liver Cancer (2020) 9:640–62. doi: 10.1159/000511001 PMC7768150 33442538

[6] Montella L Palmieri G Addeo R Del Prete S . Hepatocellular Carcinoma: Will Novel Targeted Drugs Really Impact the Next Future? World J Gastroenterol (2016) 22:6114–26. doi: 10.3748/wjg.v22.i27.6114 PMC4945973 27468204

[7] Taketomi A . Clinical Trials of Antiangiogenic Therapy for Hepatocellular Carcinoma. Int J Clin Oncol (2016) 21:213–8. doi: 10.1007/s10147-016-0966-0 26899258

[8] Nie J Lin B Zhou M Wu L Zheng T . Role of Ferroptosis in Hepatocellular Carcinoma. J Cancer Res Clin Oncol (2018) 144:2329–37. doi: 10.1007/s00432-018-2740-3 PMC11813439 30167889

[9] Liu L Cao Y Chen C Zhang X McNabola A Wilkie D . Sorafenib Blocks the RAF/MEK/ERK Pathway, Inhibits Tumor Angiogenesis, and Induces Tumor Cell Apoptosis in Hepatocellular Carcinoma Model PLC/PRF/5. Cancer Res (2006) 66:11851–8. doi: 10.1158/0008-5472 17178882

[10] Keating GM Santoro A . Sorafenib: A Review of Its Use in Advanced Hepatocellular Carcinoma. Drugs (2009) 69:223–40. doi: 10.1007/s11523-017-0484-7 19228077

[11] Llovet JM Ricci S Mazzaferro V Hilgard P Gane E Blanc JF . Sorafenib in Advanced Hepatocellular Carcinoma. N Engl J Med (2008) 359:378–90. doi: 10.1056/NEJMoa0708857 18650514

[12] Kudo M Finn RS Qin S Han KH Ikeda K Piscaglia F . Lenvatinib Versus Sorafenib in First-Line Treatment of Patients With Unresectable Hepatocellular Carcinoma: A Randomised Phase 3 Non-Inferiority Trial. Lancet (2018) 391:1163–73. doi: 10.1016/S0140-6736(18)30207-1 29433850

[13] Finn RS Qin S Ikeda M Galle PR Ducreux M Kim TY . Atezolizumab Plus Bevacizumab in Unresectable Hepatocellular Carcinoma. N Engl J Med (2020) 382:1894–905. doi: 10.1056/NEJMoa1915745 32402160

[14] Ren Z Xu J Bai Y Xu A Cang S Du C . Sintilimab Plus a Bevacizumab Biosimilar (IBI305) Versus Sorafenib in Unresectable Hepatocellular Carcinoma (ORIENT-32): A Randomised, Open-Label, Phase 2-3 Study. Lancet Oncol (2021) 22:977–90. doi: 10.1016/S1470-2045(21)00252-7 34143971

[15] Higgins JP Altman DG Gøtzsche PC Jüni P Moher D Oxman AD . The Cochrane Collaboration’s Tool for Assessing Risk of Bias in Randomised Trials. BMJ (2011) 343:d5928. doi: 10.1136/bmj.d5928 22008217 PMC3196245

[16] Atkins D Best D Briss PA Eccles M Falck-Ytter Y Flottorp S . Grading Quality of Evidence and Strength of Recommendations. BMJ (2004) 328:1490. doi: 10.1136/bmj.328.7454.1490 15205295 PMC428525

[17] Guyatt GH Oxman AD Santesso N Helfand M Vist G Kunz R . GRADE Guidelines: 12. Preparing Summary of Findings Tables-Binary Outcomes. J Clin Epidemiol (2013) 66:158–72. doi: 10.1016/j.jclinepi.2012.01.012 22609141

[18] Rücker G Schwarzer G . Reduce Dimension or Reduce Weights? Comparing Two Approaches to Multi-Arm Studies in Network Meta-Analysis. Stat Med (2014) 33:4353–69. doi: 10.1002/sim.6236 24942211

[19] Cheng AL Kang YK Chen Z Tsao CJ Qin S Kim JS . Efficacy and Safety of Sorafenib in Patients in the Asia-Pacific Region With Advanced Hepatocellular Carcinoma: A Phase III Randomised, Double-Blind, Placebo-Controlled Trial. Lancet Oncol (2009) 10:25–34. doi: 10.1016/S1470-2045(08)70285-7 19095497

[20] Cheng AL Kang YK Lin DY Park JW Kudo M Qin S . Sunitinib Versus Sorafenib in Advanced Hepatocellular Cancer: Results of a Randomized Phase III Trial. J Clin Oncol (2013) 31:4067–75. doi: 10.1200/JCO.2012.45.8372 24081937

[21] Johnson PJ Qin S Park JW Poon RT Raoul JL Philip PA . Brivanib Versus Sorafenib as First-Line Therapy in Patients With Unresectable, Advanced Hepatocellular Carcinoma: Results From the Randomized Phase III BRISK-FL Study. J Clin Oncol (2013) 31:3517–24. doi: 10.1200/JCO.2012.48.4410 23980084

[22] Cheng AL Kang YK He AR Lim HY Ryoo BY Hung CH . Investigators’ Study Group. Safety and Efficacy of Tigatuzumab Plus Sorafenib as First-Line Therapy in Subjects With Advanced Hepatocellular Carcinoma: A Phase 2 Randomized Study. J Hepatol (2015) 63:896–904. doi: 10.1016/j.jhep.2015.06.001 26071796

[23] Cainap C Qin S Huang WT Chung IJ Pan H Cheng Y . Linifanib Versus Sorafenib in Patients With Advanced Hepatocellular Carcinoma: Results of a Randomized Phase III Trial. J Clin Oncol (2015) 33:172–9. doi: 10.1200/JCO.2013.54.3298 PMC4279237 25488963

[24] Zhu AX Rosmorduc O Evans TR Ross PJ Santoro A Carrilho FJ . SEARCH: A Phase III, Randomized, Double-Blind, Placebo-Controlled Trial of Sorafenib Plus Erlotinib in Patients With Advanced Hepatocellular Carcinoma. J Clin Oncol (2015) 33:559–66. doi: 10.1200/JCO.2013.53.7746 25547503

[25] Kudo M Ryoo BY Lim HY Kim DY Okusaka T Ikeda M . Resminostat and Sorafenib Combination Therapy for Advanced Hepatocellular Carcinoma in Patients Previously Untreated With Systemic Chemotherapy. J Clin Oncol (2017) 35:252. doi: 10.1200/JCO.2017.35.4_suppl.252

[26] Abou-Alfa GK Shi Q Knox JJ Kaubisch A Niedzwiecki D Posey J . Assessment of Treatment With Sorafenib Plus Doxorubicin vs Sorafenib Alone in Patients With Advanced Hepatocellular Carcinoma: Phase 3 CALGB 80802 Randomized Clinical Trial. JAMA Oncol (2019) 5:1582–8. doi: 10.1001/jamaoncol.2019.2792 PMC6735405 31486832

[27] Yau T Park JW Finn RS Cheng AL Mathurin P Edeline J . CheckMate 459: A Randomized, Multi-Center Phase III Study of Nivolumab (NIVO) vs Sorafenib (SOR) as First-Line (1L) Treatment in Patients (Pts) With Advanced Hepatocellular Carcinoma (aHCC). Ann Oncol (2019) 30:874–5. doi: 10.1093/annonc/mdz394.029

[28] Assenat E Pageaux GP Thézenas S Peron JM Bécouarn Y Seitz JF . Sorafenib Alone vs. Sorafenib Plus GEMOX as 1st-Line Treatment for Advanced HCC: The Phase II Randomised PRODIGE 10 Trial. Br J Cancer (2019) 120:896–902. doi: 10.1038/s41416-019-0443-4 30944458 PMC6734663

[29] Qin S Bi F Gu S Bai Y Chen Z Wang Z . Donafenib Versus Sorafenib in First-Line Treatment of Unresectable or Metastatic Hepatocellular Carcinoma: A Randomized, Open-Label, Parallel-Controlled Phase II-III Trial. J Clin Oncol (2021) 29:JCO2100163. doi: 10.1200/JCO.21.00163 PMC8445562 34185551

[30] Gu W Tong Z . Sorafenib in the Treatment of Patients With Hepatocellular Carcinoma (HCC) and Microvascular Infiltration: A Systematic Review and Meta-Analysis. J Int Med Res (2020) 48:300060520946872. doi: 10.1177/0300060520946872 32815430 PMC7444130

[31] Al-Salama ZT Syed YY Scott LJ . Lenvatinib: A Review in Hepatocellular Carcinoma. Drugs (2019) 79:665–74. doi: 10.1007/s40265-019-01116-x 30993651

[32] Chiew Woon L Joycelyn Jie Xin L Su Pin C . Nivolumab for the Treatment of Hepatocellular Carcinoma. Expert Opin Biol Ther (2020) 20:687–93. doi: 10.1080/14712598.2020.1749593 32249635

[33] Weisberg E Meng C Case AE Sattler M Tiv HL Gokhale PC . Comparison of Effects of Midostaurin, Crenolanib, Quizartinib, Gilteritinib, Sorafenib and BLU-285 on Oncogenic Mutants of KIT, CBL and FLT3 in Haematological Malignancies. Br J Haematol (2019) 187:488–501. doi: 10.1111/bjh.16092 31309543 PMC7887860

[34] Boehm S Rothermundt C Hess D Joerger M . Antiangiogenic Drugs in Oncology: A Focus on Drug Safety and the Elderly – A Mini-Review. Gerontology (2010) 56:303–9. doi: 10.1159/000262450 19940466

[35] Bruix J Qin S Merle P Granito A Huang YH Bodoky G . Regorafenib for Patients With Hepatocellular Carcinoma Who Progressed on Sorafenib Treatment (RESORCE): A Randomised, Double-Blind, Placebo-Controlled, Phase 3 Trial. Lancet (2017) 389:56–66. doi: 10.1016/S0140-6736(16)32453-9 27932229

[36] Facciorusso A Abd El Aziz MA Sacco R . Efficacy of Regorafenib in Hepatocellular Carcinoma Patients: A Systematic Review and Meta-Analysis. Cancers (Basel) (2019) 12:36. doi: 10.3390/cancers12010036 PMC7017079 31877664

[37] Cabanillas ME Takahashi S . Managing the Adverse Events Associated With Lenvatinib Therapy in Radioiodine-Refractory Differentiated Thyroid Cancer. Semin Oncol (2019) 46:57–64. doi: 10.1053/j.seminoncol.2018.11.004 30685073

[38] Hao Z Wang P . Lenvatinib in Management of Solid Tumors. Oncologist (2020) 25:e302–10. doi: 10.1634/theoncologist PMC7011622 32043789

[39] Zhao Y Zhang YN Wang KT Chen L . Lenvatinib for Hepatocellular Carcinoma: From Preclinical Mechanisms to Anti-Cancer Therapy. Biochim Biophys Acta Rev Cancer (2020) 1874:188391. doi: 10.1016/j.bbcan.2020.188391 32659252

[40] Ficht X Iannacone M . Immune Surveillance of the Liver by T Cells. Sci Immunol (2020) 5:eaba2351. doi: 10.1126/sciimmunol.aba2351 32887842

[41] Horst AK Neumann K Diehl L Tiegs G . Modulation of Liver Tolerance by Conventional and Nonconventional Antigen-Presenting Cells and Regulatory Immune Cells. Cell Mol Immunol (2016) 13:277–92. doi: 10.1038/cmi.2015.112 PMC4856800 27041638

[42] Hellmann MD Paz-Ares L Bernabe Caro R Zurawski B Kim SW Carcereny Costa E . Nivolumab Plus Ipilimumab in Advanced Non-Small-Cell Lung Cancer. N Engl J Med (2019) 381:2020–31. doi: 10.1056/NEJMoa1910231 31562796

[43] Motzer RJ Tannir NM McDermott DF Arén Frontera O Melichar B Choueiri TK . CheckMate 214 Investigators. Nivolumab Plus Ipilimumab Versus Sunitinib in Advanced Renal-Cell Carcinoma. N Engl J Med (2018) 378:1277–90. doi: 10.1056/NEJMoa1712126 PMC5972549 29562145

[44] Larkin J Hodi FS Wolchok JD . Combined Nivolumab and Ipilimumab or Monotherapy in Untreated Melanoma. N Engl J Med (2015) 373:1270–1. doi: 10.1056/NEJMc1509660 26398076

[45] Calderaro J Rousseau B Amaddeo G Mercey M Charpy C Costentin C . Programmed Death Ligand 1 Expression in Hepatocellular Carcinoma: Relationship With Clinical and Pathological Features. Hepatology (2016) 64:2038–46. doi: 10.1002/hep.28710 27359084

[46] El-Khoueiry AB Sangro B Yau T Crocenzi TS Kudo M Hsu C . Nivolumab in Patients With Advanced Hepatocellular Carcinoma (CheckMate 040): An Open-Label, Non-Comparative, Phase 1/2 Dose Escalation and Expansion Trial. Lancet (2017) 389:2492–502. doi: 10.1016/S0140-6736(17)31046-2 PMC7539326 28434648

[47] Xu J Shen J Gu S Zhang Y Wu L Wu J . Camrelizumab in Combination With Apatinib in Patients With Advanced Hepatocellular Carcinoma (RESCUE): A Nonrandomized, Open-Label, Phase II Trial. Clin Cancer Res (2021) 27:1003–11. doi: 10.1158/1078-0432 33087333

[48] Sangro B Gomez-Martin C de la Mata M Iñarrairaegui M Garralda E Barrera P . A Clinical Trial of CTLA-4 Blockade With Tremelimumab in Patients With Hepatocellular Carcinoma and Chronic Hepatitis C. J Hepatol (2013) 59:81–8. doi: 10.1016/j.jhep.2013.02.022 23466307

[49] Yau T Kang YK Kim TY El-Khoueiry AB Santoro A Sangro B . Efficacy and Safety of Nivolumab Plus Ipilimumab in Patients With Advanced Hepatocellular Carcinoma Previously Treated With Sorafenib: The CheckMate 040 Randomized Clinical Trial. JAMA Oncol (2020) 6:e204564. doi: 10.1001/jamaoncol.2020.4564 33001135 PMC7530824

[50] Abd El Aziz MA Facciorusso A Nayfeh T Saadi S Elnaggar M Cotsoglou C . Immune Checkpoint Inhibitors for Unresectable Hepatocellular Carcinoma. Vaccines (Basel) (2020) 8:616. doi: 10.3390/vaccines8040616 PMC7712941 33086471

[51] Hoy SM . Sintilimab: First Global Approval. Drugs (2019) 79:341–6. doi: 10.1007/s40265-019-1066-z 30742278

