# Supplementary material for: First-Line Systemic Treatment Strategies for Unresectable Hepatocellular Carcinoma: A Systematic Review and Network Meta-Analysis of Randomized Clinical Trials
Source: Front Oncol. 2021 Dec 24;11:771045. doi: 10.3389/fonc.2021.771045 (PMC8739799; doi:10.3389/fonc.2021.771045)
Supplement: Supplementary file 1 [file DataSheet_1.docx]

Supplementary Figure 1. Network plot depicting all the evidence

Supplementary Figure 2. Potential bias assessment and quality of included studies using the Cochrane Risk Bias Tool

Supplementary Figure 3A. The cumulative ranking plot with the surface under the cumulative ranking curve (SUCRA) and P score for the outcome of OS

Supplementary Figure 3B. The cumulative ranking plot with the surface under the cumulative ranking curve (SUCRA) and P score for the outcome of PFS

Supplementary Figure 3C. The cumulative ranking plot with the surface under the cumulative ranking curve (SUCRA) and P score for the outcome of AEs

Supplementary Figure 3D. The cumulative ranking plot with the surface under the cumulative ranking curve (SUCRA) and P score for the outcome of grade 3-5 AEs

Supplementary Figure 4A. the funnel plot for OS

Supplementary Figure 4B. the funnel plot for PFS

Supplementary Figure 4C. the funnel plot for AEs

Supplementary Figure 4D. the funnel plot for SAEs

Supplementary Table1 Certainty of Evidence Table (GRADE). First line treatment - Overall survival

Supplementary Table 2 Certainty of Evidence Table (GRADE). First line treatment - Progression free survival

Supplementary Table 3 Certainty of Evidence Table (GRADE). First line treatment - Adverse events

Supplementary Table 4 Certainty of Evidence Table (GRADE). First line treatment - Serious adverse events


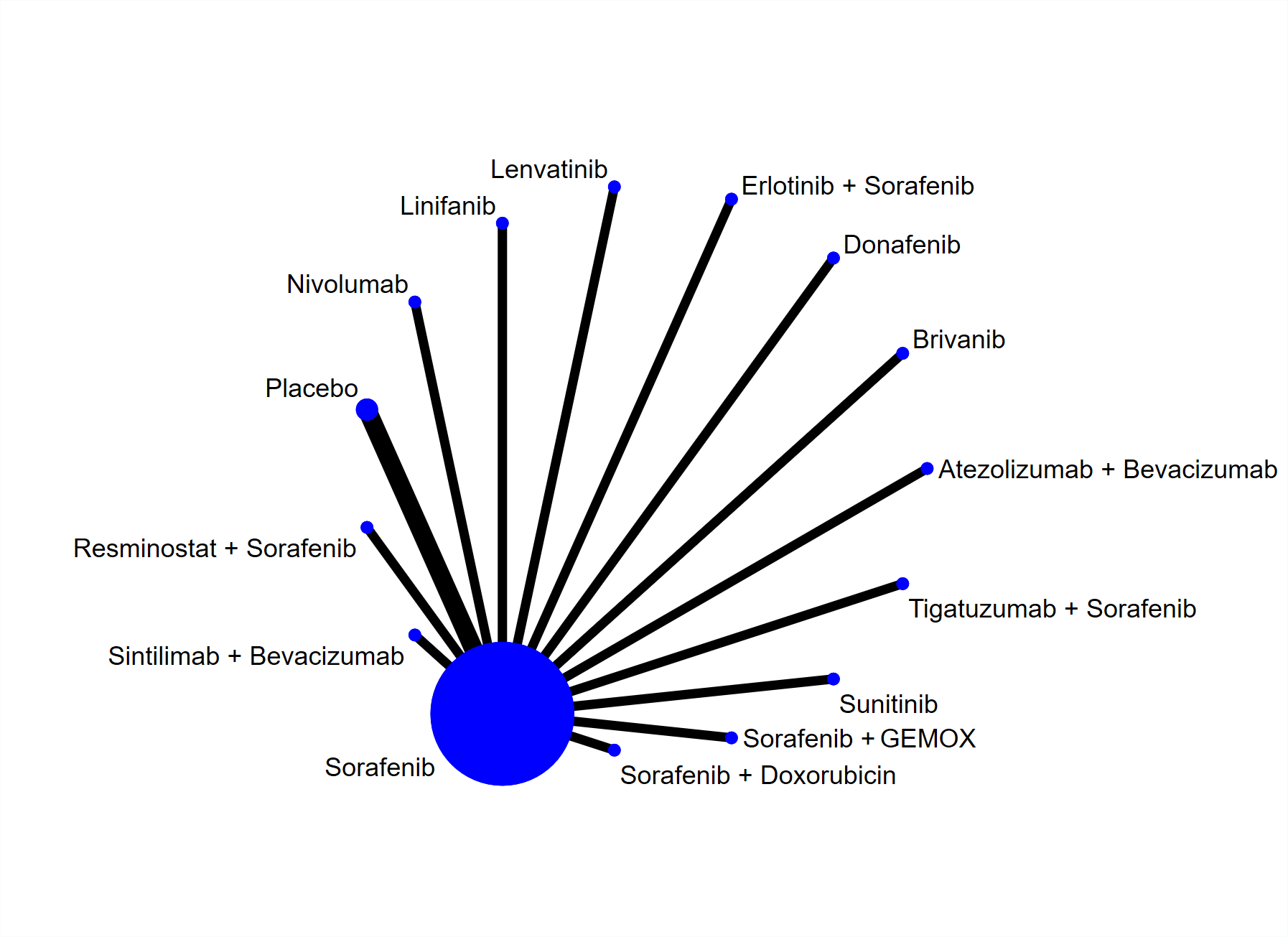


Supplementary Figure 1. Network plot depicting all the evidence


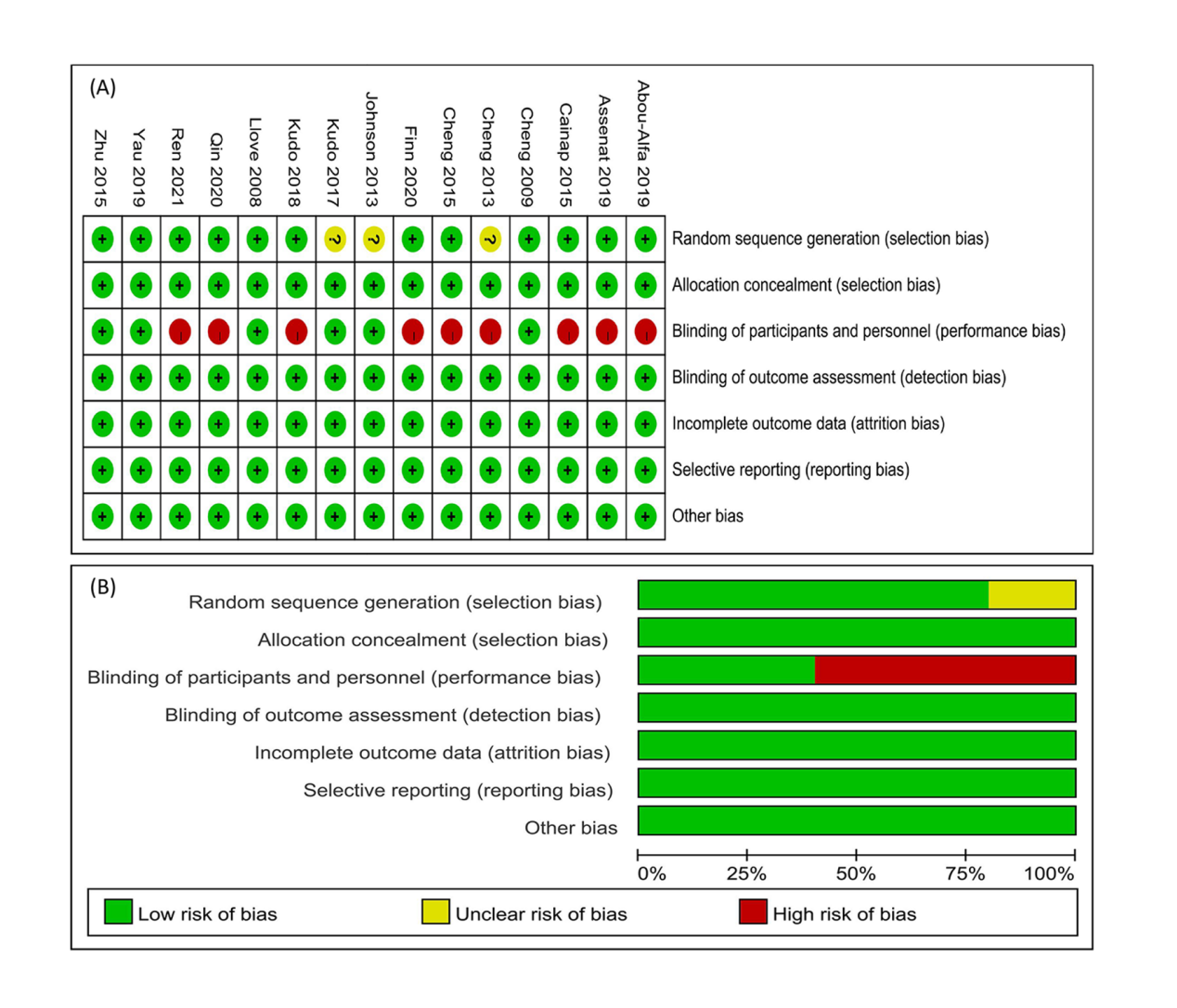


Supplementary Figure 2. Potential bias assessment and quality of included studies using the Cochrane Risk Bias Tool


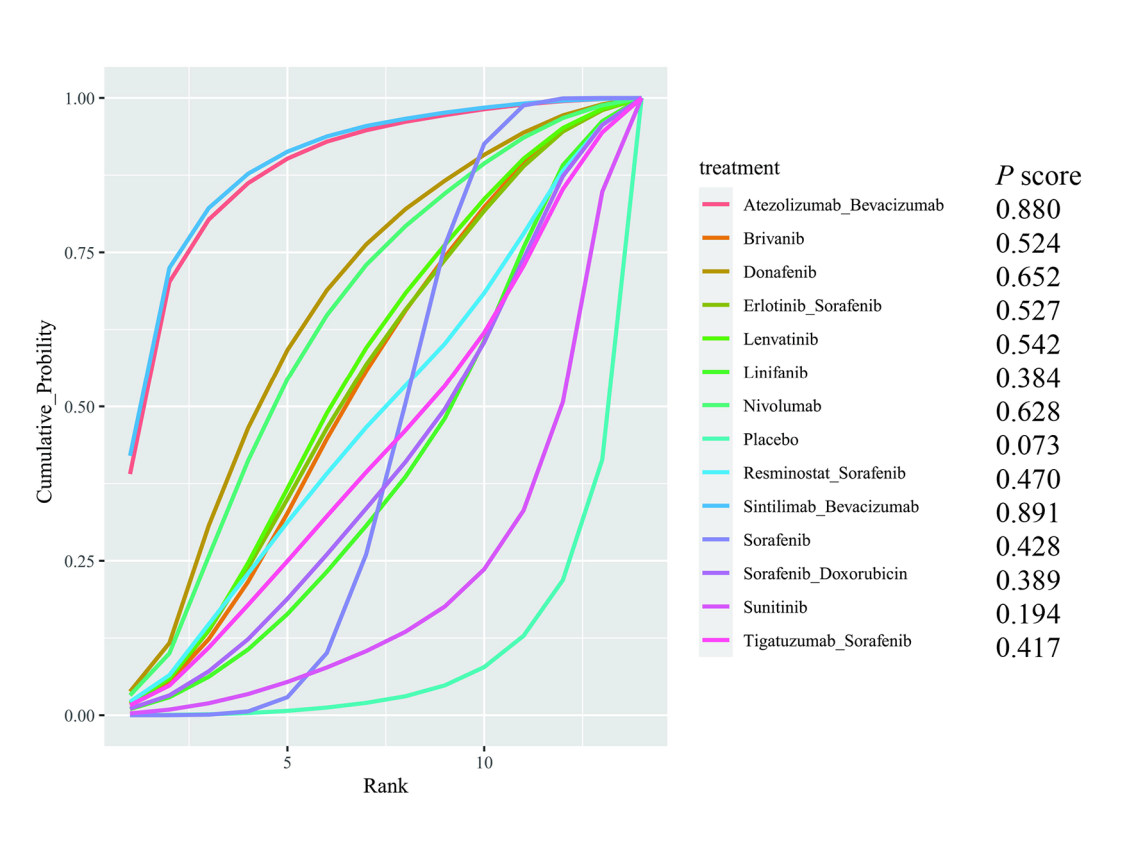


Supplementary Figure 3A. The cumulative ranking plot with the surface under the cumulative ranking curve (SUCRA) and P score for the outcome of OS


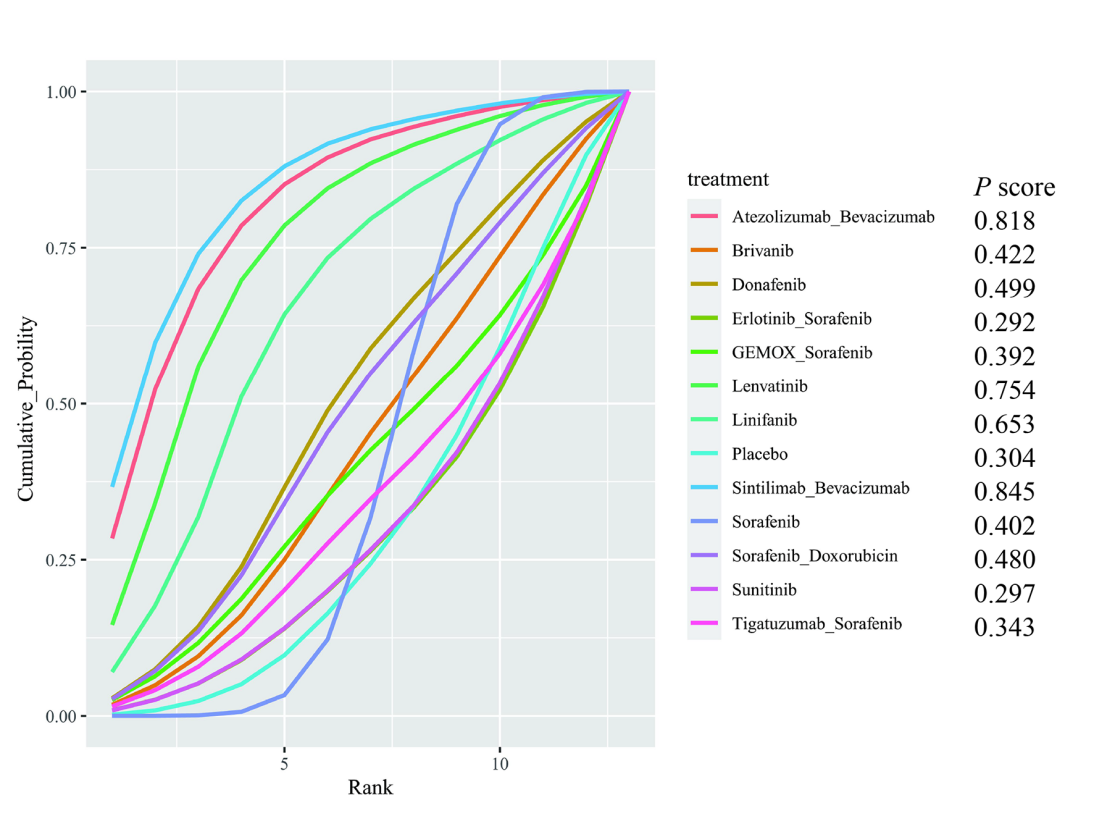


Supplementary Figure 3B. The cumulative ranking plot with the surface under the cumulative ranking curve (SUCRA) and P score for the outcome of PFS


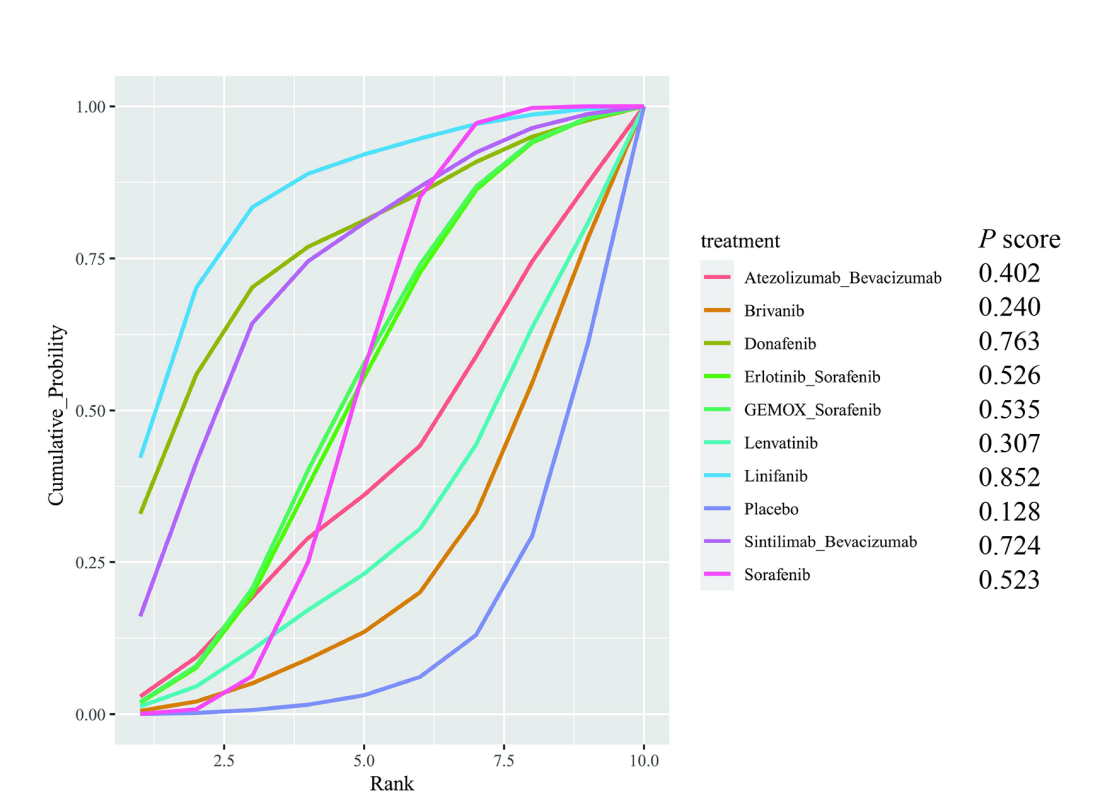


Supplementary Figure 3C. The cumulative ranking plot with the surface under the cumulative ranking curve (SUCRA) and P score for the outcome of AEs


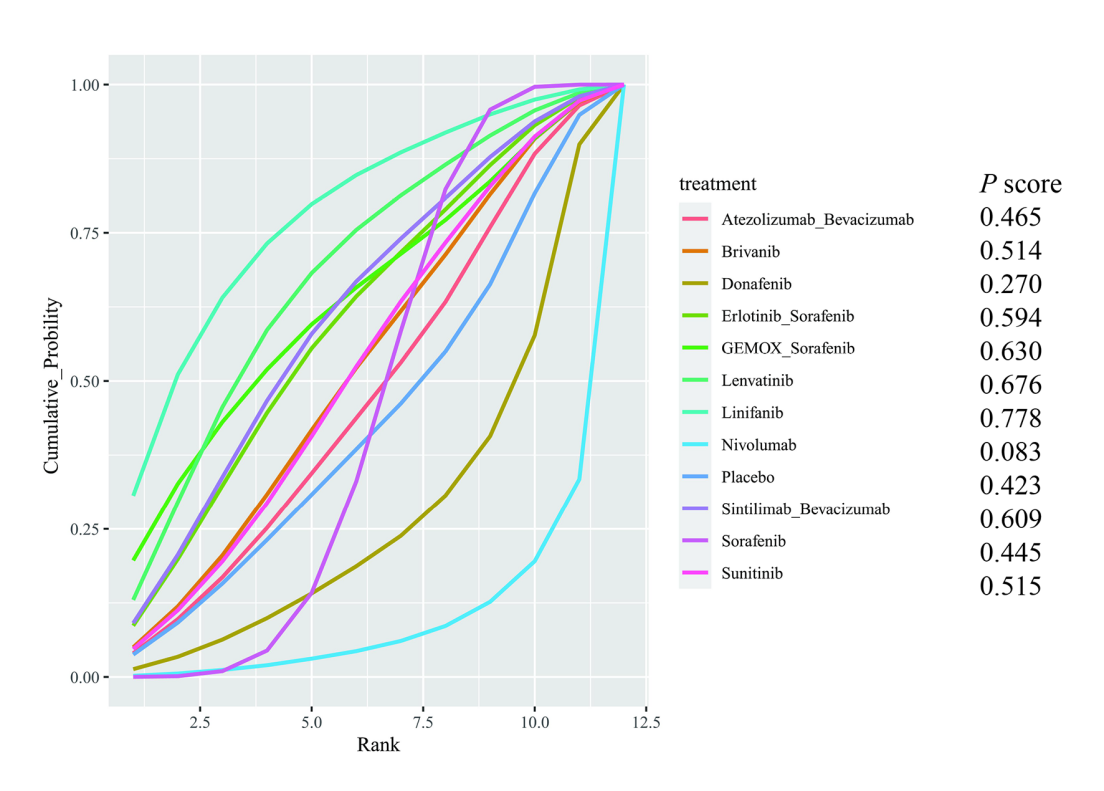


Supplementary Figure 3D. The cumulative ranking plot with the surface under the cumulative ranking curve (SUCRA) and P score for the outcome of SAEs


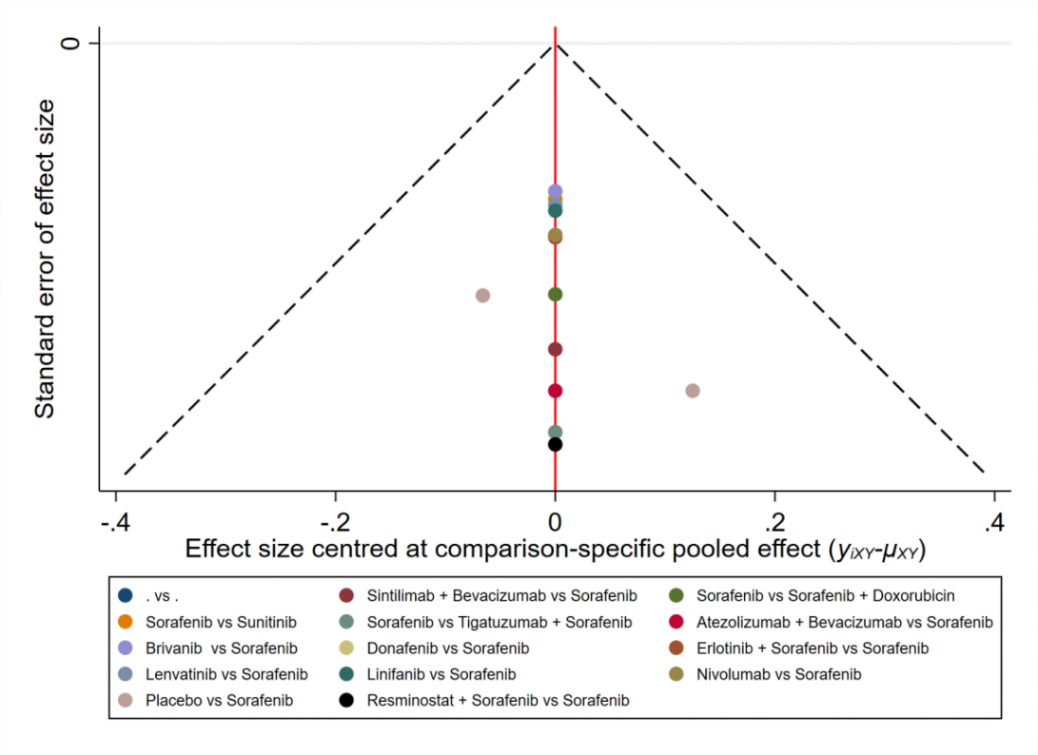


Egger’s p=0.372

Supplementary Figure 4A. the funnel plot for OS


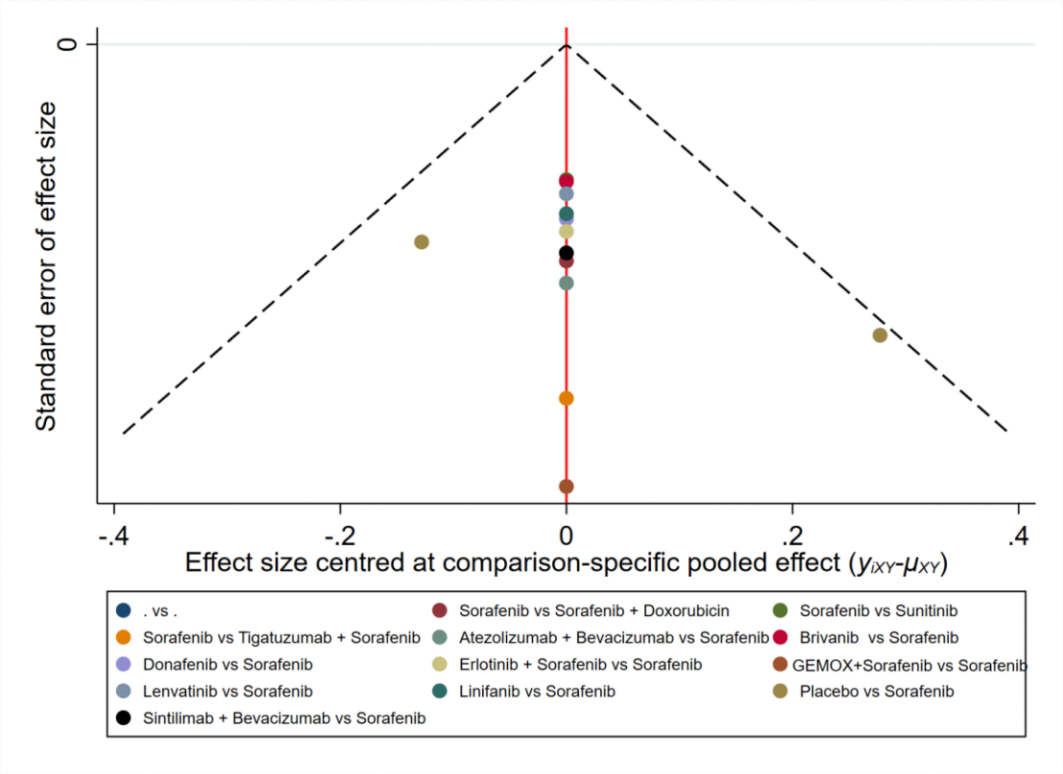


Egger’s p=0.565

Supplementary Figure 4B. the funnel plot for PFS


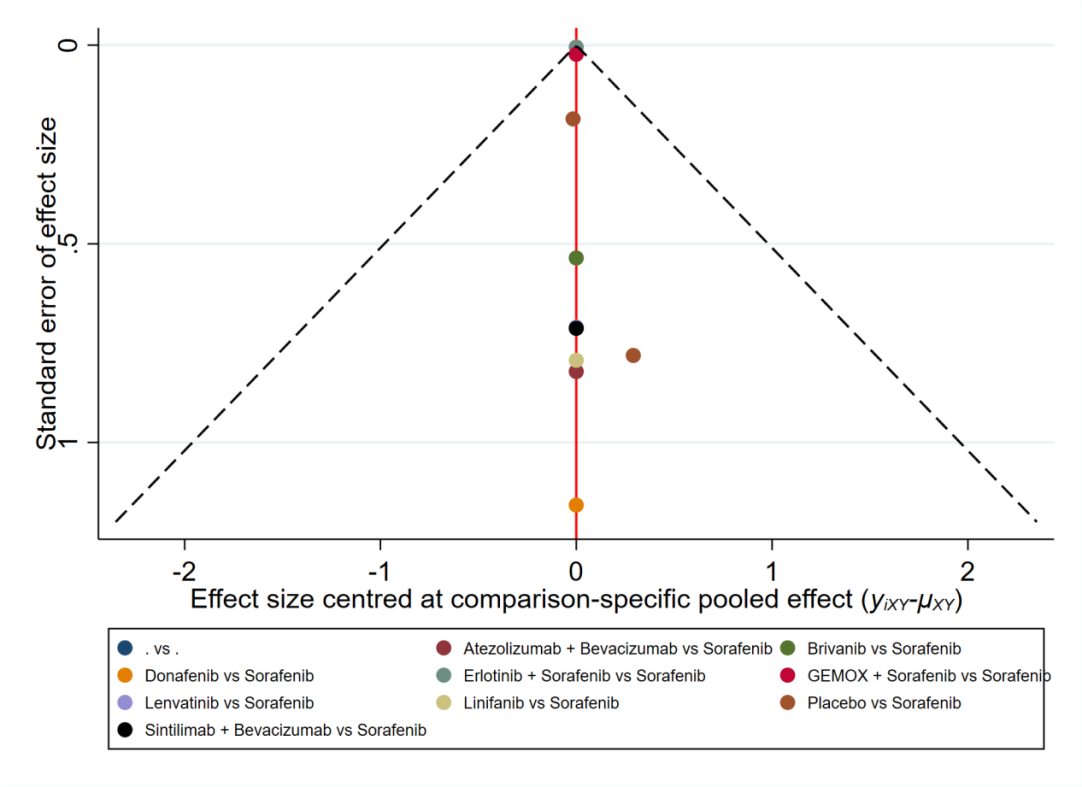


Egger’s p=0.163

Supplementary Figure 4C. the funnel plot for AEs


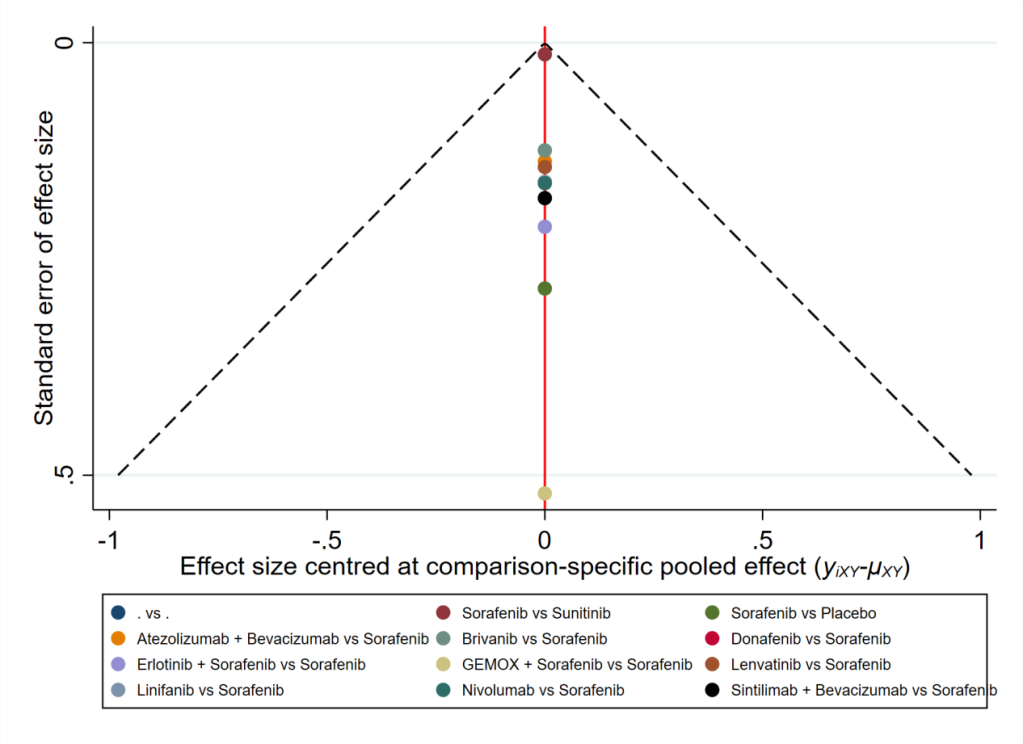


Egger’s p=0.533

Supplementary Figure 4D. the funnel plot for grade 3-5 AEs

**Supplementary Table1 Certainty of Evidence Table (GRADE). First line treatment - Overall survival**

| **Certainty assessment** | | | | | | | | | | | | **№ of patients** | | **Effect** | | **Certainty** | **Importance** | | | | |
| --- | --- | --- | --- | --- | --- | --- | --- | --- | --- | --- | --- | --- | --- | --- | --- | --- | --- | --- | --- | --- | --- |
| **№ of studies** | | **Study design** | | | | | **Risk of bias** | **Inconsistency** | **Indirectness** | **Imprecision** | **Other considerations** | **First line**  **OS** | **Risk of**  **Death**  **in**  **Control** | **Relative (95% CI)** | **Absolute (95% CI)** |  |  |  |  |  |  |
| **Atezolizumab + Bevacizumab vs Sorafenib - Overall survival** | | | | | | | | | | | | | | | | | | | | | |
| 1 | | randomised trials | | | | | not serious | not serious | not serious | not serious | none |  |  | **HR 0.58** (0.42 to 0.79) |  | ⨁⨁⨁⨁ High | CRITICAL | | | | |
| **Donafenib vs Sorafenib - Overall survival** | | | | | | | | | | | | | | | | | | | | | |
| 1 | | randomised trials | | | | | not serious | not serious | not serious | not serious | none |  |  | **HR 0.83** (0.69 to 0.98) |  | ⨁⨁⨁⨁ High | CRITICAL | | | | |
| **Erlotinib + Sorafenib vs Sorafenib - Overall survival** | | | | | | | | | | | | | | | | | | | | | |
| 1 | | randomised trials | | | | | not serious | not serious | not serious | serious | none |  |  | **HR 0.93** (0.78 to 1.11) |  | ⨁⨁⨁◯ Moderate | CRITICAL | | | | |
| **Lenvatinib vs Sorafenib - Overall survival** | | | | | | | | | | | | | | | | | | | | | |
| 1 | | randomised trials | | | | | not serious | not serious | not serious | serious | none |  |  | **HR 0.92** (0.79 to 1.06) |  | ⨁⨁⨁◯ Moderate | CRITICAL | | | | |
| **Linifanib vs Sorafenib - Overall survival** | | | | | | | | | | | | | | | | | | | | | |
| 1 | | | randomised trials | | | | not serious | not serious | not serious | serious | none |  |  | **HR 1.05** (0.90 to 1.22) |  | ⨁⨁⨁◯ Moderate | | CRITICAL | | | |
| **Nivolumab vs Sorafenib - Overall survival** | | | | | | | | | | | | | | | | | | | | | |
| 1 | | | randomised trials | | | | not serious | not serious | not serious | serious | none |  |  | **HR 0.85** (0.72 to 1.02) |  | ⨁⨁⨁◯ Moderate | | CRITICAL | | | |
| **Resminostat + Sorafenib vs Sorafenib - Overall survival** | | | | | | | | | | | | | | | | | | | | | |
| 1 | | | randomised trials | | | | not serious | not serious | not serious | serious | none |  |  | **HR 0.98** (0.68 to 1.41) |  | ⨁⨁⨁◯ Moderate | | CRITICAL | | | |
| **Sintilimab + Bevacizumab vs Sorafenib - Overall survival** | | | | | | | | | | | | | | | | | | | | | |
| 1 | | | | randomised trials | | | not serious | not serious | not serious | not serious | none |  |  | **HR 0.57** (0.43 to 0.75) |  | ⨁⨁⨁⨁ High | | | CRITICAL | | |
| **Sorafenib + Doxorubicin vs Sorafenib - Overall survival** | | | | | | | | | | | | | | | | | | | | | |
| 1 | | | | | randomised trials | | not serious | not serious | not serious | serious | none |  |  | **HR 1.05** (0.72 to 1.02) |  | ⨁⨁⨁◯ Moderate | | | CRITICAL | | |
| **Sorafenib vs Brivanib - Overall survival** | | | | | | | | | | | | | | | | | | | | | |
| 2 | | | | | | randomised trials | not serious | not serious | not serious | serious | none |  |  | **HR 1.07** (0.94 to 1.23) |  | ⨁⨁⨁◯ Moderate | | | | CRITICAL | |
| **Sorafenib vs Placebo - Overall survival** | | | | | | | | | | | | | | | | | | | | | |
| 1 | randomised trials | | | | | | not serious | not serious | not serious | not serious | none |  |  | **HR 0.65** (0.53 to 0.78) |  | ⨁⨁⨁⨁ High | | | | | CRITICAL |
| **Sunitinib vs Sorafenib - Overall survival** | | | | | | | | | | | | | | | | | | | | | |
| 1 | randomised trials | | | | | | not serious | not serious | not serious | not serious | none |  |  | **HR 1.30** (1.13 to 1.5) |  | ⨁⨁⨁⨁ High | | | | | CRITICAL |
| **Tigatuzumab + Sorafenib vs Sorafenib - Overall survival** | | | | | | | | | | | | | | | | | | | | | |
| 1 | randomised trials | | | | | | not serious | not serious | not serious | serious | none |  |  | **HR 1.03** (0.72 to 1.46) |  | ⨁⨁⨁◯ Moderate | | | | | CRITICAL |

# **OS: overall survival; CI:** confidence interval; **HR:** hazard ratio

**Supplementary Table 2 Certainty of Evidence Table (GRADE). First line treatment - Progression free survival**

| **Certainty assessment** | | | | | | | | | | | | **№ of patients** | | **Effect** | | **Certainty** | **Importance** | | | | |
| --- | --- | --- | --- | --- | --- | --- | --- | --- | --- | --- | --- | --- | --- | --- | --- | --- | --- | --- | --- | --- | --- |
| **№ of studies** | | **Study design** | | | | | **Risk of bias** | **Inconsistency** | **Indirectness** | **Imprecision** | **Other considerations** | **First line**  **PFS** | **Risk of**  **Death**  **in**  **Control** | **Relative (95% CI)** | **Absolute (95% CI)** |  |  |  |  |  |  |
| **Atezolizumab + Bevacizumab vs Sorafenib - Progression free survival** | | | | | | | | | | | | | | | | | | | | | |
| 1 | | randomised trials | | | | | not serious | not serious | not serious | not serious | none |  |  | **HR 0.59** (0.47 to 0.76) |  | ⨁⨁⨁⨁ High | CRITICAL | | | | |
| **Donafenib vs Sorafenib - Progression free survival** | | | | | | | | | | | | | | | | | | | | | |
| 1 | | randomised trials | | | | | not serious | not serious | not serious | serious | none |  |  | **HR 0.91** (0.76 to 1.08) |  | ⨁⨁⨁◯ Moderate | CRITICAL | | | | |
| **Erlotinib + Sorafenib vs Sorafenib - Progression free survival** | | | | | | | | | | | | | | | | | | | | | |
| 1 | | randomised trials | | | | | not serious | not serious | not serious | serious | none |  |  | **HR 1.14** (0.94 to 1.37) |  | ⨁⨁⨁◯ Moderate | CRITICAL | | | | |
| **Lenvatinib vs Sorafenib - Progression free survival** | | | | | | | | | | | | | | | | | | | | | |
| 1 | | randomised trials | | | | | not serious | not serious | not serious | not serious | none |  |  | **HR 0.66** (0.57 to 0.77) |  | ⨁⨁⨁⨁ High | CRITICAL | | | | |
| **Linifanib vs Sorafenib - Progression free survival** | | | | | | | | | | | | | | | | | | | | | |
| 1 | | | randomised trials | | | | not serious | not serious | not serious | not serious | none |  |  | **HR 0.76** (0.64 to 0.90) |  | ⨁⨁⨁⨁ High | | CRITICAL | | | |
| **Sintilimab + Bevacizumab vs Sorafenib - Progression free survival** | | | | | | | | | | | | | | | | | | | | | |
| 1 | | | randomised trials | | | | not serious | not serious | not serious | not serious | none |  |  | **HR 0.56** (0.46 to 0.70) |  | ⨁⨁⨁⨁ High | | CRITICAL | | | |
| **Sorafenib + Doxorubicin vs Sorafenib - Progression free survival** | | | | | | | | | | | | | | | | | | | | | |
| 1 | | | randomised trials | | | | not serious | not serious | not serious | serious | none |  |  | **HR 0.93** (0.75 to 1.16) |  | ⨁⨁⨁◯ Moderate | | CRITICAL | | | |
| **Sorafenib vs Brivanib - Progression free survival** | | | | | | | | | | | | | | | | | | | | | |
| 1 | | | | randomised trials | | | not serious | not serious | not serious | serious | none |  |  | **HR 1.01** (0.88 to 1.16) |  | ⨁⨁⨁◯ Moderate | | | CRITICAL | | |
| **Sorafenib vs GEMOX+Sorafenib - Progression free survival** | | | | | | | | | | | | | | | | | | | | | |
| 1 | | | | | randomised trials | | not serious | not serious | not serious | serious | none |  |  | **HR 0.97** (0.62 to 1.51) |  | ⨁⨁⨁◯ Moderate | | | CRITICAL | | |
| **Sorafenib vs Placebo - Progression free survival** | | | | | | | | | | | | | | | | | | | | | |
| 2 | | | | | | randomised trials | not serious | not serious | not serious | serious | none |  |  | **HR 0.95** (0.81 to 1.12) |  | ⨁⨁⨁◯ Moderate | | | | CRITICAL | |
| **Sunitinib vs Sorafenib - Progression free survival** | | | | | | | | | | | | | | | | | | | | | |
| 1 | randomised trials | | | | | | not serious | not serious | not serious | serious | none |  |  | **HR 1.13** (0.99 to 1.30) |  | ⨁⨁⨁◯ Moderate | | | | | CRITICAL |
| **Tigatuzumab + Sorafenib vs Sorafenib - Progression free survival** | | | | | | | | | | | | | | | | | | | | | |
| 1 | randomised trials | | | | | | not serious | not serious | not serious | serious | none |  |  | **HR 1.09** (0.76 to 1.55) |  | ⨁⨁⨁◯ Moderate | | | | | CRITICAL |

# **PFS: progression free survival; CI:** confidence interval; **HR:** hazard ratio

**Supplementary Table 3 Certainty of Evidence Table (GRADE). First line treatment - Adverse events**

| **Certainty assessment** | | | | | | | | | | **№ of patients** | | **Effect** | | **Certainty** | **Importance** | | |
| --- | --- | --- | --- | --- | --- | --- | --- | --- | --- | --- | --- | --- | --- | --- | --- | --- | --- |
| **№ of studies** | **Study design** | | | | **Risk of bias** | **Inconsistency** | **Indirectness** | **Imprecision** | **Other considerations** | **First line**  **AEs** | **Risk of**  **Death**  **in**  **Control** | **Relative (95% CI)** | **Absolute (95% CI)** |  |  |  |  |
| **Atezolizumab + Bevacizumab vs Sorafenib - Adverse events** | | | | | | | | | | | | | | | | | |
| 1 | randomised trials | | | | not serious | not serious | not serious | serious | none |  |  | **OR 0.70** (0.14 to 3.50) |  | ⨁⨁⨁◯ Moderate | CRITICAL | | |
| **Donafenib vs Sorafenib - Adverse events** | | | | | | | | | | | | | | | | | |
| 1 | randomised trials | | | | not serious | not serious | not serious | serious | none |  |  | **OR 3.03** (0.31 to 29.25) |  | ⨁⨁⨁◯ Moderate | CRITICAL | | |
| **Erlotinib + Sorafenib vs Sorafenib - Adverse events** | | | | | | | | | | | | | | | | | |
| 1 | randomised trials | | | | not serious | not serious | not serious | serious | none |  |  | **OR 1.00** (0.99 to 1.01) |  | ⨁⨁⨁◯ Moderate | CRITICAL | | |
| **Lenvatinib vs Sorafenib - Adverse events** | | | | | | | | | | | | | | | | | |
| 1 | randomised trials | | | | not serious | not serious | not serious | serious | none |  |  | **OR 0.50** (0.12 to 2.00) |  | ⨁⨁⨁◯ Moderate | CRITICAL | | |
| **Linifanib vs Sorafenib - Adverse events** | | | | | | | | | | | | | | | | | |
| 1 | | randomised trials | | | not serious | not serious | not serious | serious | none |  |  | **OR 3.97** (0.84 to 18.82) |  | ⨁⨁⨁◯ Moderate | | CRITICAL | |
| **Sintilimab + Bevacizumab vs Sorafenib - Adverse events** | | | | | | | | | | | | | | | | | |
| 1 | | randomised trials | | | not serious | not serious | not serious | serious | none |  |  | **0R 2.08** (0.51 to 8.40) |  | ⨁⨁⨁◯ Moderate | | CRITICAL | |
| **Sorafenib vs GEMOX + Sorafenib - Adverse events** | | | | | | | | | | | | | | | | | |
| 1 | | randomised trials | | | not serious | not serious | not serious | serious | none |  |  | **OR 0.98** (0.93 to 1.02) |  | ⨁⨁⨁◯ Moderate | | CRITICAL | |
| **Sorafenib vs Brivanib - Adverse events** | | | | | | | | | | | | | | | | | |
| 1 | | | randomised trials | | not serious | not serious | not serious | serious | none |  |  | **OR 2.43** (0.85 to 6.95) |  | ⨁⨁⨁◯ Moderate | | | CRITICAL |
| **Sorafenib vs Placebo - Adverse events** | | | | | | | | | | | | | | | | | |
| 2 | | | | randomised trials | not serious | not serious | not serious | serious | none |  |  | **OR 3.57** (0.99 to 11.11) |  | ⨁⨁⨁◯ Moderate | | | CRITICAL |

# **AEs: adverse events; CI: confidence interval; OR: odd ratios**

**Supplementary Table 4 Certainty of Evidence Table (GRADE). First line treatment - Serious adverse events**

| **Certainty assessment** | | | | | | | | | | **№ of patients** | | **Effect** | | **Certainty** | **Importance** | | |
| --- | --- | --- | --- | --- | --- | --- | --- | --- | --- | --- | --- | --- | --- | --- | --- | --- | --- |
| **№ of studies** | **Study design** | | | | **Risk of bias** | **Inconsistency** | **Indirectness** | **Imprecision** | **Other considerations** | **First line**  **SAEs** | **Risk of**  **Death**  **in**  **Control** | **Relative (95% CI)** | **Absolute (95% CI)** |  |  |  |  |
| **Atezolizumab + Bevacizumab vs Sorafenib - Serious adverse events** | | | | | | | | | | | | | | | | | |
| 1 | randomised trials | | | | not serious | not serious | not serious | serious | none |  |  | **OR 1.01** (0.68 to 1.17) |  | ⨁⨁⨁◯ Moderate | CRITICAL | | |
| **Donafenib vs Sorafenib - Serious adverse events** | | | | | | | | | | | | | | | | | |
| 1 | randomised trials | | | | not serious | not serious | not serious | not serious | none |  |  | **OR 0.65** (0.47 to 0.89) |  | ⨁⨁⨁⨁ High | CRITICAL | | |
| **Erlotinib + Sorafenib vs Sorafenib - Serious adverse events** | | | | | | | | | | | | | | | | | |
| 1 | randomised trials | | | | not serious | not serious | not serious | serious | none |  |  | **OR 1.28** (0.85 to 1.95) |  | ⨁⨁⨁◯ Moderate | CRITICAL | | |
| **Lenvatinib vs Sorafenib - Serious adverse events** | | | | | | | | | | | | | | | | | |
| 1 | randomised trials | | | | not serious | not serious | not serious | not serious | none |  |  | **OR 1.51** (1.13 to 2.00) |  | ⨁⨁⨁⨁ High | CRITICAL | | |
| **Linifanib vs Sorafenib - Serious adverse events** | | | | | | | | | | | | | | | | | |
| 1 | | randomised trials | | | not serious | not serious | not serious | not serious | none |  |  | **OR 1.94** (1.41 to 2.66) |  | ⨁⨁⨁⨁ High | | CRITICAL | |
| **Nivolumab vs Sorafenib - Serious adverse events** | | | | | | | | | | | | | | | | | |
| 1 | | randomised trials | | | not serious | not serious | not serious | not serious | none |  |  | **0R 0.30** (0.21 to 0.41) |  | ⨁⨁⨁⨁ High | | CRITICAL | |
| **Sintilimab + Bevacizumab vs Sorafenib - Serious adverse events** | | | | | | | | | | | | | | | | | |
| 1 | | randomised trials | | | not serious | not serious | not serious | serious | none |  |  | **OR 1.32** (0.93 to 1.88) |  | ⨁⨁⨁◯ Moderate | | CRITICAL | |
| **Sorafenib vs GEMOX + Sorafenib - Serious adverse events** | | | | | | | | | | | | | | | | | |
| 1 | | | randomised trials | | not serious | not serious | not serious | serious | none |  |  | **OR0.69** (0.25 to 1.91) |  | ⨁⨁⨁◯ Moderate | | | CRITICAL |
| **Sorafenib vs Brivanib - Serious adverse events** | | | | | | | | | | | | | | | | | |
| 1 | | | | randomised trials | not serious | not serious | not serious | serious | none |  |  | **OR0.91** (0.71 to 1.16) |  | ⨁⨁⨁◯ Moderate | | | CRITICAL |
| **Sorafenib vs Placebo - Serious adverse events** | | | | | | | | | | | | | | | | | |
| 1 | | | randomised trials | | not serious | not serious | not serious | serious | none |  |  | **OR 1.10** (0.63 to 1.92) |  | ⨁⨁⨁◯ Moderate | | | CRITICAL |
| **Sunitinib vs Sorafenib - Serious adverse events** | | | | | | | | | | | | | | | | | |
| 1 | | | | randomised trials | not serious | not serious | not serious | not serious | none |  |  | **OR 1.10** (1.07 to 1.13) |  | ⨁⨁⨁⨁ High | | | CRITICAL |

# **SAEs: serious adverse events; CI: confidence interval; OR: odd ratios**
